# Severe Dietary Energy Restriction for Compensated Cirrhosis Due to Metabolic Dysfunction‐Associated Steatotic Liver Disease: A Randomised Controlled Trial

**DOI:** 10.1002/jcsm.13783

**Published:** 2025-04-24

**Authors:** Dimitrios A. Koutoukidis, Susan A. Jebb, Jeremy W. Tomlinson, Ferenc E. Mozes, Michael Pavlides, Miriam Lacharie, Francesca Saffioti, Paul Aveyard, Jeremy F. Cobbold

**Affiliations:** ^1^ Nuffield Department of Primary Care Health Sciences University of Oxford Oxford UK; ^2^ NIHR Oxford Biomedical Research Centre Oxford UK; ^3^ Oxford Centre for Diabetes, Endocrinology and Metabolism, Radcliffe Department of Medicine University of Oxford Oxford UK; ^4^ Oxford Centre for Clinical Magnetic Resonance Research, Radcliffe Department of Medicine University of Oxford Oxford UK; ^5^ Department of Gastroenterology and Hepatology John Radcliffe Hospital, Oxford University Hospitals NHS Foundation Trust Oxford UK

**Keywords:** cirrhosis, diet, magnetic resonance, MASLD, weight loss

## Abstract

**Background:**

Compensated cirrhosis due to metabolic dysfunction‐associated steatotic liver disease (CC‐MASLD) increases morbidity and mortality risk but has no aetiology‐specific treatment. We investigated the safety and efficacy signals of severe energy restriction.

**Methods:**

In this randomised controlled trial, adults with CC‐MASLD and obesity in a tertiary hepatology centre were randomised 2:1 to receive one‐to‐one remote dietetic support with a low‐energy (880 kcal/day, 80 g protein/day) total diet replacement programme for 12 weeks and stepped food reintroduction for another 12 weeks or standard of care (SoC). Given the exploratory nature of the study, three pre‐defined co‐primary outcomes were used to assess safety and efficacy signals: severe increases in liver biochemistry, changes in iron‐corrected T1, and changes in liver stiffness on magnetic resonance elastography. Changes in liver steatosis on magnetic resonance imaging, physical performance based on the physical performance test and liver frailty index, and changes in fat‐free mass were secondary outcomes. Magnetic resonance outcomes were assessed blind.

**Results:**

Between February 2022 and September 2023, 17 participants (36% female, median [IQR] age 58 [7.5] years) were randomised to SoC (*n* = 6) or intervention (*n* = 11). The trial stopped earlier than planned due to slow recruitment rate. 91% and 94% of participants completed the intervention and attended the 24‐week follow‐up, respectively. Compared with the SoC, the between‐group weight change in the intervention was −11.9 kg (95% CI: −17.2, −6.6, *p* < 0.001) at 24 weeks. Liver biochemistry markers (alanine transaminase, aspartate transaminase, and total bilirubin) were stable in everyone throughout the trial. Iron‐corrected T1 and steatosis significantly reduced (−149.9 ms [95% CI −258.1, −41.7, *p* = 0.01] and −6% [95% CI −11.3, −0.6, *p* = 0.03], respectively). There were no between‐group differences in changes in liver stiffness (0.2 kPa [95% CI −1.1, 1.6]), the physical performance test (1.5 points [95% CI −1.9 to 4.9], *p* = 0.70) or the liver frailty index (0 [95% CI −0.6 to 0.6], *p* = 0.97). Compared with SoC, absolute fat‐free mass reduced (−3.2 kg [95% CI −6 to −0.3], *p* = 0.04) but relative fat‐free mass as percentage of total body weight increased (5.4% [95% CI 0.5 to 10.3], *p* = 0.046). No participant met the pre‐defined safety criteria for enhanced observation or intervention discontinuation. There was no between‐group differences in changes in cardiovascular markers and no evidence of hepatic decompensation or serious adverse events.

**Conclusions:**

Severe energy restriction appears a safe option to achieve significant weight loss and reduce liver fat without adverse effects in people with CC‐MASLD. A larger study is needed to confirm these findings.

**Clinical Trial Registration:**

ISRCTN13053035, prospectively registered, overall study status: closed.

## 1 Introduction

Compensated cirrhosis due to metabolic dysfunction‐associated steatotic liver disease (CC‐MASLD) is a progressive and severe form of MASLD. CC‐MASLD cases are estimated to more than double by 2030 in many countries worldwide [1]. This is estimated to increase decompensated cirrhosis and liver cancer cases, and consequently increase the need for liver transplantation [2, 3]. Compared with early‐stage MASLD with no or mild fibrosis, people with CC‐MASLD have lower quality of life, along with a 42‐fold and 6‐fold increased risk of liver‐related and all‐cause mortality, respectively [4, 5, 6, 7]. This is expected to add significantly to the costs for the healthcare system [8]. Most patients also have obesity, which is independently linked with disease progression and cardiovascular risk [9].

Currently, there is no aetiology‐specific pharmacotherapy to treat CC‐MASLD, but clinical guidelines recommend that clinicians offer advice to patients with MASLD lose weight with a 7–10% weight loss target [10]. However, the evidence for effectiveness is limited because advice to lose weight alone usually leads to small weight loss that is unlikely to impact on liver outcomes [11]. We previously showed that dietary energy restriction leading to rapid and substantial weight loss has a favourable safety profile and significantly reduced the severity of liver disease in people with metabolic dysfunction‐associated steatohepatitis (MASH) and liver fibrosis stage 2–3 [12]. This approach may also have a positive impact on people with CC‐MASLD, although clinical guidelines do not specifically recommend substantial weight loss in this population, given concerns that it might cause liver dysfunction or decompensation [13] together with concerns for potential compromises in physical function through reductions in muscle mass in those with sarcopenia. We investigated the signals of safety and efficacy of dietary energy restriction in people with CC‐MASLD, specifically looking for evidence of any harms.

## 2 Methods

### 2.1 Trial Design

This was a prospectively registered parallel randomised controlled trial with blinded outcome assessors (ISRCTN13053035). The authors had access to the study data and reviewed and approved the final manuscript.

### 2.2 Participants

We recruited adults with a histological or clinical diagnosis of CC‐MASLD and a body mass index (BMI) ≥ 30 kg/m^2^. The main exclusion criteria were evidence of alternative or co‐existing aetiologies for cirrhosis, history of sustained harmful alcohol intake and alcohol intake of ≥18 units for females and ≥26 units for males over the previous 7 days, no evidence of hepatic decompensation (jaundice, ascites, hepatic encephalopathy, or variceal haemorrhage), weight loss of 10% or more since diagnostic biopsy or, if biopsy was not present, within the last 6 months, and unstable dose of medication for type 2 diabetes in the 3 months prior to screening. Detailed inclusion and exclusion criteria specifying, among others, the definition for CC‐MASLD, are in the appended trial protocol. Since the beginning of the study, the criteria were slightly amended to widen the participant pool, minimise unnecessary exclusions, and facilitate recruitment, as detailed in the protocol amendment history.

Participants were recruited from the tertiary hepatology centre at the Oxford University Hospitals National Health Service (NHS) Foundation Trust. Interested participants who were identified by the Royal Berkshire NHS Foundation Trust hepatology clinic were referred to Oxford for the study. All the participants provided written informed consent.

### 2.3 Interventions

#### 2.3.1 Low‐Energy Total Diet Replacement with Behavioural Support

Participants were supported to replace their usual food with a nutritionally replete package of four meal replacement products a day providing 880 kcal/day (Optifast®, Nestle Health Science) for 16 weeks. Between weeks 17 and 22, the participants gradually reduced their consumption of the replacement products and replaced them with food‐based meals in line with the healthy eating recommendations. In weeks 23–24, they only consumed food‐based meals. Throughout the 24 weeks, all participants received support from a registered dietitian (Oviva, UK) over the phone or via a mobile app for approximately 15 minutes weekly or 30 minutes fortnightly. The pre‐specified criteria for enhanced observation and intervention discontinuation are available in the attached protocol.

#### 2.3.2 Standard of Care

Participants in the standard of care (SoC) group received brief advice from their hepatologist on healthy eating.

### 2.4 Co‐Primary Outcomes

The study had three co‐primary outcomes to assess signals of safety and efficacy: (a) severe changes in liver biochemistry (alanine transaminase (ALT), aspartate transaminase (AST), total bilirubin) as defined in the appended intervention discontinuation algorithm; (b) changes in iron‐corrected T1 (cT1), a marker of fibro‐inflammation, at 24 weeks; and (c) changes in liver stiffness measured with magnetic resonance elastography (MRE), a marker of liver fibrosis, at 24 weeks. We hypothesised that there would be no severe deterioration in biochemistry, the cT1 would reduce, and that the MRE‐measured liver stiffness would not worsen compared with the SoC.

### 2.5 Secondary Outcomes

Secondary outcomes included changes in

- additional markers of cirrhosis and liver disease severity: liver stiffness by transient elastography, liver steatosis measured with magnetic resonance imaging (MRI) proton density fat fraction and controlled attenuation parameter, enhanced liver fibrosis (ELF) score, the United Kingdom Model for End‐Stage Liver Disease (UKELD) [14], Child‐Pugh score [15], alkaline phosphatase (ALP), INR, prothrombin time, and conjugated bilirubin.
- physical performance: objectively measured physical performance test based on evaluating 9 activities (e.g. sit to stand, grip strength, walking 15 m, progressive Romberg test, stair climbing) [16] and liver frailty index [17] and
- anthropometric: weight and fat‐free mass.

### 2.6 Exploratory Outcomes

Changes in cardiometabolic markers (blood pressure, haemoglobin A1c [HbA1c] and lipid profile) and adjustments in the number and dose of relevant medications.

### 2.7 Procedures

The procedures that the participants underwent for the assessment of the outcomes are detailed in the supplementary file.

### 2.8 Criteria for Progression to a Definitive Trial

Pre‐specified criteria for progression to a definitive trial included recruitment (randomising ≥25% of eligible participants), adherence (≥60% of participants in the intervention group who commenced the programme having lost ≥10% of their weight at 16 weeks), retention (≥85% of participants completing the 24‐week visit), and safety based on adverse and serious adverse reactions.

### 2.9 Sample Size

No sample size calculation was performed for the severe changes in biochemistry, as these were the main safety signals. With 24 participants (*n* = 16 intervention, *n* = 8 control), the study was powered to detect a between‐group difference of 100 ms in cT1 with 90% power at the 5% level assuming an SD for cT1 of 70 ms and a dropout rate of 10% at 24 weeks. This was based on (a) the correlation between an 88 ms difference in cT1 with changes in histological endpoints [18], (b) the observed change of 130 ms in cT1 in our previous trial of weight loss in metabolic dysfunction‐associated steatohepatitis with fibrosis stage 2–3 [12], and (c) the expectation of a more conservative between‐group difference of 100 ms here given the more advanced disease state.

### 2.10 Randomisation

Following the baseline visit, the participants were individually randomised with a 2:1 allocation to receive either the intervention or SoC through minimisation with a 20% random element. The two stratified variables were BMI (≥/< 35 kg/m^2^) and type 2 diabetes (yes/no), as these are prognostic factors for clinical outcomes.

Randomisation took place using a validated minimisation software (MinimPY [19]) run by an independent researcher who was informed by the chief investigator of the stratification values following each baseline visit. Allocation concealment was achieved by concealing the processes of recruitment, screening, and eligibility assessment from the researcher performing the randomisation centrally, performing the randomisation following the baseline visit, not carrying out baseline visits for additional participants during the run‐in period between the baseline visit of a participant and their randomisation.

### 2.11 Blinding

The MRI/MRE operators and independent assessors of the MRI/MRE scans were blinded to the intervention allocation, time point of each scan, and clinical variables. The blood‐based markers were also assessed blind to the above at the hospital laboratory.

### 2.12 Long‐Term Follow‐Up

We extracted data on long‐term follow‐up (approximately 1 year and 1.5 years from baseline) from hospital records based on routine care appointments. These included weight, ALT, liver stiffness, summaries from liver ultrasound reports, and clinical assessments.

### 2.13 Statistical Analysis

We followed our prospectively registered and pre‐specified statistical analysis plan. We analysed the data with an intention‐to‐treat principle including all participants randomised to their original group with available data. We did not impute missing data. All models were adjusted for the stratification factors using a single four‐level categorical variable. For outcomes assessed at ≥3 timepoints, data were analysed with mixed effects models with the participant and the stratification variable as random effects and intervention allocation, timepoint and a multiplicative interaction term between allocation and timepoint as fixed effects. The interaction term provided the intervention effect, i.e. the mean differences in change from baseline between groups with 95% CIs. For outcomes assessed at 2 timepoints, data were analysed with a mixed effects model adjusted for baseline value as a fixed effect and the stratification factor as a random effect. Where any mixed model was (almost) singular, we re‐run it with the stratification variable as fixed effect. As per our statistical plan, we graphically present but did not statistically analyse blood‐based liver markers. For the per‐protocol analysis, we compared participants in the intervention group who had lost ≥10% of their weight at 16 weeks against those in SoC. Given that the sample size was smaller than expected, our planned descriptive reporting of subgroups was not meaningful. For long‐term follow‐up, we imputed weight data with linear regression between follow‐up time points if data at further follow‐up were available. Data were analysed in RStudio v2023.09.1 and R [20].

## 3 Results

**FIGURE 1:**
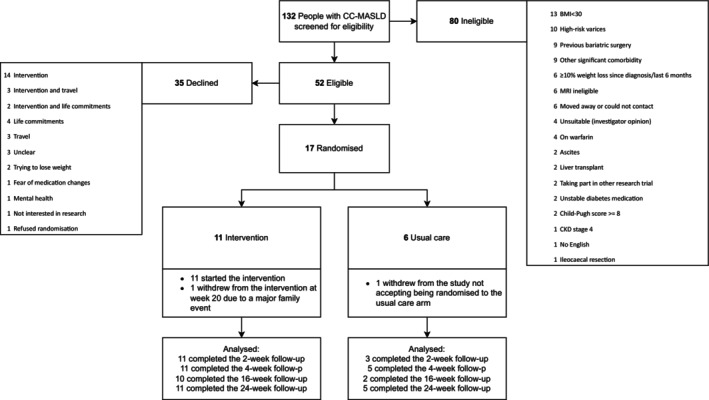
CONSORT diagram.

Between February 2022 and September 2023, 52 potentially eligible patients were approached to take part in the study. Of those, 35 (67%) declined with the primary reason being unwillingness to commit to the intervention and 17 (compared with the original recruitment target of 24) were randomised to intervention (*n* = 11) or SoC (*n* = 6) groups (Figure 1). Ten (91%) participants completed the intervention, and 15 had complete data at 24 weeks (*n* = 10 and *n* = 5 in intervention and SoC, respectively). Baseline characteristics are shown in Table S1. Two patients had a clinical diagnosis of CC‐MASLD and 15 patients had a histological diagnosis. Of those with a histological diagnosis, the median MASLD activity score was 5 and everyone had at least one point in the fat and ballooning components indicating the presence of metabolic dysfunction associated steatohepatitis. (Figure S1).

### 3.1 Markers of Liver Disease Severity for Signals of Safety and Efficacy

**FIGURE 2:**
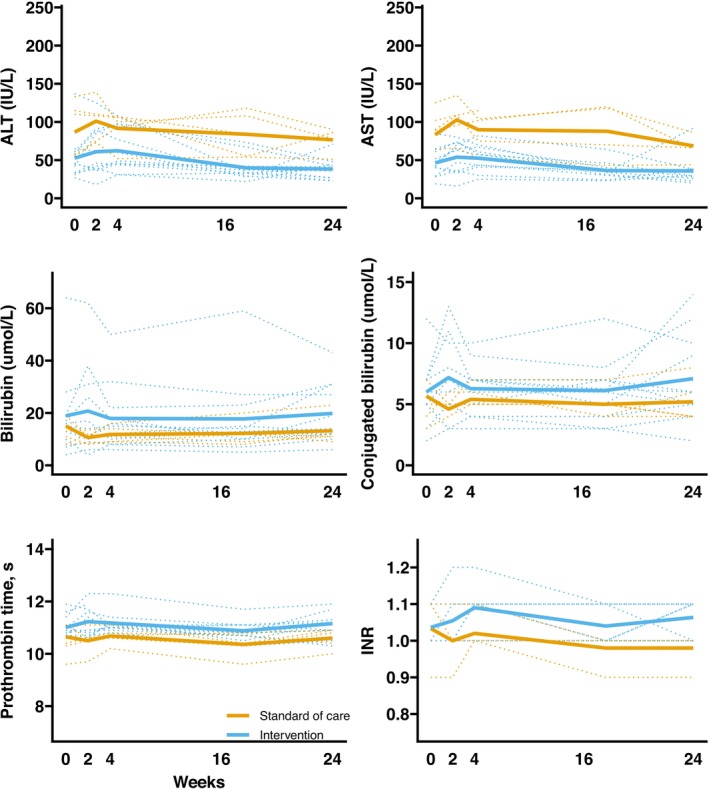
Individual (dotted) and average (solid) absolute changes by group in ALT, AST, total bilirubin, conjugated bilirubin, prothrombin time, and INR.

Liver biochemistry remained stable throughout the trial in all participants. There were no severe changes in ALT, AST or total bilirubin levels and no signs or symptoms of hepatic decompensation; therefore, none of the participants met the pre‐specified criteria for entering the enhanced observation protocol or intervention discontinuation. As pre‐specified in the statistical analysis plan, we did not analyse changes in ALT, AST, bilirubin, prothrombin time, and INR, but present these descriptively in Table 1 and Figure 2. These plots showed no concerning signals in any participant. There was no between‐group differences in changes in ALP, the Child‐Pugh score or UKELD score at any time point (Figure S2).

**TABLE 1:** Baseline mean (SD) and mean change from baseline (95% CI) at 24 weeks for each of the markers of cirrhosis and liver disease severity by group and between‐group difference.

Marker	Intervention		Standard of care		Adjusted difference (95% CI)
	Baseline	Change at 24 weeks	Baseline	Change at 24 weeks	
ALT, IU/L	52.6 (30.7)	−14.4 (34.4)	86.7 (36.7)	−17.4 (33.8)	
AST, IU/L	46.4 (18.9)	−10.5 (23.7)	83.2 (33.1)	−25.4 (7.9)	
Bilirubin, μmol/L	18.9 (16.2)	0.9 (9.3)	15 (8.8)	1.4 (2.3)	
Conjugated bilirubin, μmol/L	6 (2.6)	1.1 (3.2)	5.7 (2.5)	0.4 (0.9)	
INR	1 (0.1)	0 (0)	1 (0.1)	0 (0.1)	
Prothrombin time	11 (0.4)	0.1 (0.4)	10.7 (0.7)	0.1 (0.3)	
UKELD	46.4 (2.7)	0.6 (1.5)	46.7 (2.8)	0.6 (1.2)	0.1 (−1.5 to 1.9)
Child‐Pugh score	5.3 (0.6)	−0.1 (0.3)	5 (0)	0 (0)	−0.1 (−0.5 to 0.2)
ELF score	9.5 (1.1)	0.5 (1.3)	10.8 (1.3)	−1.0 (1.3)	0.6 (−0.8 to 2.0)
cT1, ms	949.6 (171.5)	−89.8 (123.5)	1049.2 (161.8)	−32.8 (85.4)	−149.9 (−258.1 to −41.7)
Liver PDFF, %	15 (8.2)	−5.8 (4.8)	19.3 (12.9)	−2.3 (3.8)	−6 (−11.3 to −0.6)
CAP, dB/m	338.9 (31.1)	−57 (44.9)	359.7 (30.3)	9 (51.9)	−68.3 (−114.5 to −23.3)
MRE‐measured liver stiffness, kPa	5 (1.3)	−0.2 (0.8)	6 (3.1)	−0.9 (1.5)	0.2 (−1.1 to 1.6)
VCTE‐measured liver stiffness, kPa	21.4 (9.7)	−2.3 (4.9)	24.4 (12.6)	−0.6 (6.1)	−1.1 (−7.3 to 5.4)

Abbreviations: ALP, alkaline phosphatase; ALT, alanine aminotransferase; AST, aspartate aminotransferase; CAP, controlled attenuation parameter; cT1, corrected T1; ELF, enhanced liver fibrosis score; MRE, magnetic resonance elastography; PDFF, proton‐density fat fraction; UKELD, United Kingdom Model for End‐Stage Liver Disease; VCTE, vibration‐controlled transient elastography.

**FIGURE 3:**
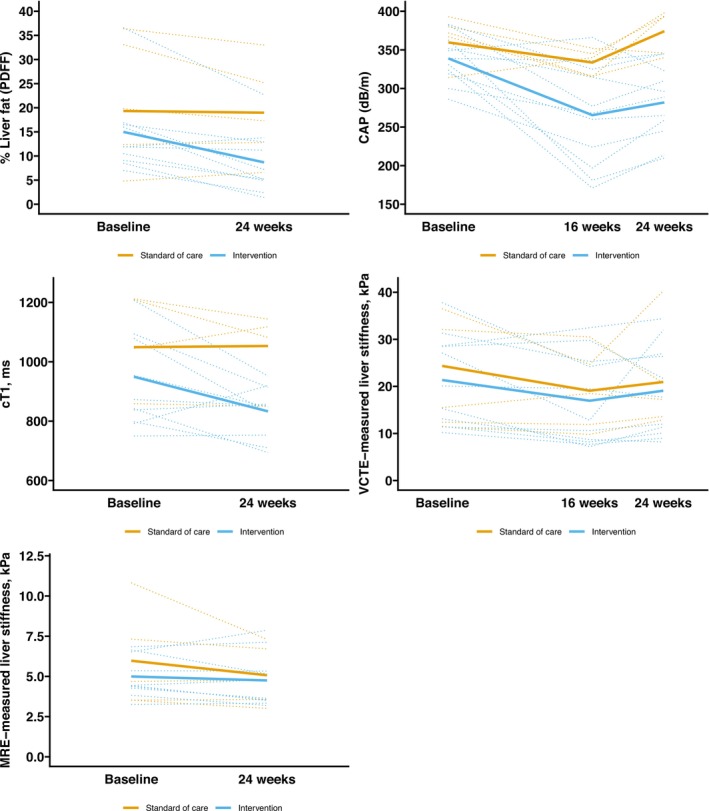
Individual (dotted) and average (solid) absolute changes by group in MRI‐proton density fat fraction (%), controlled attenuation parameter (dB/m), cT1 (ms), VCTE‐measured liver stiffness (kPa), and MRE‐measured liver stiffness (kPa). MRI, magnetic resonance imaging; MRE, magnetic resonance elastography; VCTE, vibration‐controlled transient elastography.

There was a significant reduction in cT1 (*p* = 0.01) and in both markers of liver fat (PDFF [*p* = 0.03] and CAP [*p* = 0.009]) in the intervention group compared with SoC. When the two scans for cT1 that were analysed separately were excluded from the analysis, the cT1 estimate became non‐significant (*p* = 0.07). There was no evidence of a between‐group difference in MRE‐measured and VCTE‐measured liver stiffness at 24 weeks (Figure 3).

### 3.2 Weight, Body Composition, and Physical Performance

**FIGURE 4:**
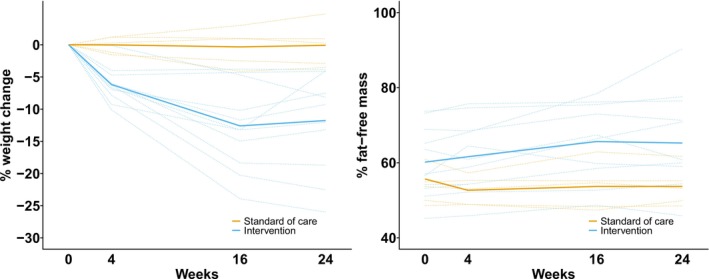
Individual (dotted) and average (solid) percentage weight change trajectories and percentage fat‐free mass by group.

Compared with SoC, participants in the intervention group lost an average −6.5 kg (−11.7 to −1.2), −13.4 kg (−18.7 to −8.0), and −11.9 kg (−17.2 to −6.6) at 4, 16, and 24 weeks, respectively. The individual weight loss trajectories are depicted in Figure 3. Eight of the 11 intervention participants (73%) lost at least 10% of their body weight at 16 weeks. Compared with SoC, absolute fat‐free mass significantly reduced at 24 weeks but the relative fat‐free mass as a percentage of total body weight increased (Table 2 and Figure 4). There was no between‐group difference in the change in physical performance test or liver frailty index at 24 weeks.

**TABLE 2:** Baseline mean (SD) and mean change from baseline (95% CI) at 24 weeks for the anthropometric, physical performance, and cardiometabolic outcome measures by group and between‐group difference.

Marker	Intervention		Standard of care		Adjusted difference (95% CI)
	Baseline	Change at 24 weeks	Baseline	Change at 24 weeks	
Weight, kg	103.6 (14.8)	−12.1 (8.3)	106.2 (9)	−0.1 (3.4)	−11.9 (−17.2 to −6.6)
Fat‐free mass, kg	61.6 (8.5)	−3.3 (3.5)	59.5 (11.5)	0 (1.8)	−3.2 (−6 to −0.3)
Fat‐free mass, %	60.2 (9.4)	5.1 (8.1)	55.7 (6.6)	0.1 (1.1)	5.4 (0.5 to 10.3)
Physical performance test^a^	34 (2.1)	−0.8 (2.3)	35.7 (0.8)	−1.6 (1.8)	1.5 (−1.9 to 4.9)
Liver frailty index^b^	3.1 (0.5)	−0.1 (0.4)	3.1 (0.3)	0 (0.3)	0 (−0.6 to 0.6)
Cholesterol to HDL ratio	3.4 (0.9)	−0.2 (0.5)	4.4 (1.2)	0 (0.6)	−0.2 (−0.9 to 0.5)
Cholesterol, mmol/L	3.7 (1)	0.1 (0.5)	5.4 (2.2)	0.3 (0.4)	−0.3 (−1.2 to 0.5)
Haemoglobin A1c, %	6.8 (1.2)	−0.8 (0.5)	7.1 (2)	−0.3 (0.7)	−0.4 (−0.9 to 0.2)
Systolic blood pressure, mmHg	127.1 (15.1)	3 (14.7)	125.2 (14.5)	0.9 (8.1)	2 (−10.9 to 15)
Diastolic blood pressure, mmHg	70.6 (6.9)	1 (8.8)	73.2 (15.8)	4.1 (8.9)	−2.6 (−10.2 to 5.3)

Abbreviation: HDL, high‐density lipoprotein.

^a^ Plausible range 0–36, with higher scores indicating better physical performance.

^b^ Plausible range: 0–7.1 with lower scores indicating lower frailty and scores <3.2 classified as robust/not frail.

### 3.3 Cardiometabolic Markers and Medication Changes

There was no significant difference in any of the measured cardiometabolic markers (Table 2). In the intervention group, all participants with type 2 diabetes at baseline had reductions in the dosage of their prescribed antidiabetic medication and these reductions were maintained at follow‐up, except for one participant whose dose was increased to baseline levels at 24 weeks. The dose of antidiabetic medication in the SoC group remained stable for one participant and was increased at 24 weeks for the other participant. All participants taking antihypertensive medications at baseline had reduction in the dose of their medication throughout the study, whereas the dosage remained stable for participants in the SoC group.

### 3.4 Per‐Protocol Analysis

In the per‐protocol analysis, we explored the impact of the intervention comparing the most adherent participants (i.e. those who lost ≥10% by 16 weeks, *n* = 8) against SoC. The between‐group difference in weight change was −14.5 kg. Participants in the intervention group lost on average more fat‐free mass by 3.4 kg, but their relative percentage of fat‐free mass increased by 6.9% compared with SoC. The between‐group reductions in PDFF, continued attenuation parameter, and cT1 were slightly more pronounced compared to the intention‐to‐treat analysis. There were no other differences compared with the intention‐to‐treat analysis, as all other markers remaining no different between the groups (Table S2).

### 3.5 Criteria for Progression to a Full Trial

The study exceeded the minimum pre‐specified criteria for recruitment (33% vs. 25% of eligible participants were recruited), adherence (73% vs. 60% of intervention participants lost at least 10% of their weight at 16 weeks), and retention (88% vs. 85% completed their final follow‐up).

### 3.6 Adverse Events

There were no decompensation events, serious events, or deaths. Ten (91%) and four (80%) of participants in the intervention and SoC groups, respectively, reported at least one adverse event. None of the participants withdrew from the trial, entered the enhanced observation, or discontinued the intervention due to adverse events. Most events were judged as mild or moderate by the participants. The most common adverse events in the intervention and SoC groups were headache (*n* = 4, 36%) and COVID‐19 (*n* = 2, 40%) (Table S3).

### 3.7 Intervention Engagement and Evaluation

Intervention engagement was high with four participants engaging fully with the dietetic support, and three of them missing one of the weekly calls each, two of them missing two calls each, and one participant missing six calls. The participant who withdrew fully engaged until that point. Nine intervention participants returned their feedback questionnaires. They all rated the programme highly with two and seven reporting the programme exceeded and met their expectations, respectively.

### 3.8 Long‐Term Follow‐Up

**FIGURE 5:**
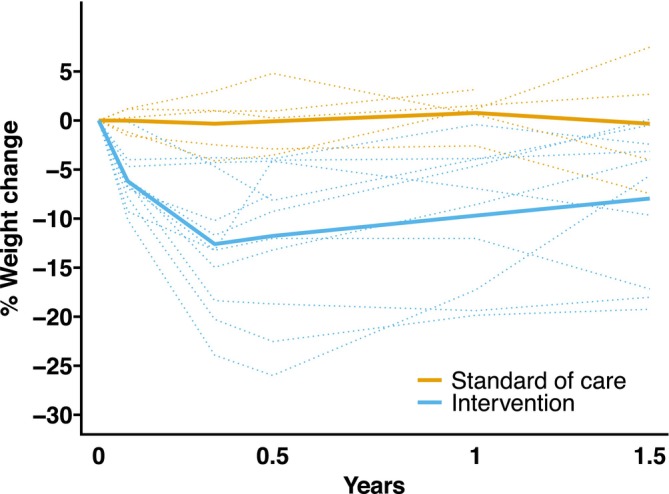
Percentage weight change at 1 and 1.5 years compared with baseline in each group.

Weight data from 10 (91%) and 5 (83%) participants from the intervention and SoC groups, respectively, were available at 1 year and from 14 patients at 1.5 years. Compared with SoC, participants in the intervention group remained at a significantly lower weight, with an average between‐group weight loss at −10.6 kg (95% CI: −16.4 to −4.8) and −8.0 kg (95% CI: −14.1 to −2.0) at 1 and 1.5 years from baseline, respectively (Figure 5). There was no evidence of worsening of ALT, and no participant with available ALT data had at least a two‐fold increase compared to their 24‐week value. There were no significant changes in liver stiffness and no evidence of hepatocellular carcinoma, disease worsening based on available liver ultrasound reports, or decompensation based on clinical assessments in any participant.

## 4 Discussion

This randomised controlled trial showed that severe dietary energy restriction through a low‐energy total diet replacement programme was acceptable to people with CC‐MASLD and led to substantial loss of weight, primarily body fat. It had a favourable safety profile with no evidence of acute liver dysfunction, hepatic decompensation, worsening of fibrosis markers, or of adverse effects on body composition and physical performance. It also led to significant reductions in liver fat and cT1, a marker of fibro‐inflammation.

The strengths of the study include its randomised design, a well‐defined cohort of people with CC‐MASLD, a comprehensive longitudinal assessment of liver disease, blinded outcome assessment, and the prospective registration of the protocol and statistical analysis plan. Despite extending our recruitment period, we were unable to reach our original recruitment target of 24 individuals because the pool of eligible participants was more limited than our prior estimates indicated, which reduced the statistical power of the study. Additional limitations of our trial include the small sample size and the lack of a follow‐up liver biopsy. Our long‐term provides reassurance based on clinical data and follow‐up supports that weight regain is only mild, albeit somewhat faster than previous estimates showing that a mild weight regain of about 2 kg/year typically occurs [21].

Our recruitment rate was 33% of eligible patients, lower than our 44% recruitment rate of eligible people with MASH and fibrosis stage 2–3 from the same hospital for a similar trial [12]. The reasons for the discrepancy remain speculative and we were unable to interview people who declined participation. The substantial weight loss with total diet replacement suggests that this is a reasonable option to achieve weight loss in patients with CC‐MASLD. Future research should explore offering also other effective weight loss programmes to cater for patient preferences, which should boost recruitment rates.

Our results of a 5.8% reduction in PDFF with 12.1% within‐group weight loss in the intervention are similar to those of a trial of semaglutide in this population, which showed a 3.5% reduction in PDFF at 48 weeks with 8.8% within‐group weight loss. Importantly, both trials have shown consistent evidence regarding the safety of weight loss in terms of liver disease with no decompensation events or worsening liver function [22]. We did not measure portal pressure, but even a 5 kg weight loss has been shown to reduce it [23]. As in the semaglutide trial, we observed no changes in markers of liver fibrosis, which indicates that weight loss in this range and short timeframe may not be sufficient to reverse advanced fibrosis. Greater weight loss of 20–30% after 2.5 years following bariatric surgery has been associated with reversal of fibrosis in people with CC‐MASLD [24]. Bariatric surgery is not a scalable option given the prevalence of CC‐MASLD and new pharmacotherapies for weight loss that can achieve sustainable weight loss of a magnitude approaching that of bariatric surgery warrant further investigation.

Concerns regarding weight loss in CC‐MASLD also related to the acceleration of muscle mass loss, sarcopenia, and loss of physical function, as these are prognostic factors in cirrhosis [25]. Loss of absolute muscle mass is unavoidable with weight loss, but our results using a low‐energy protocol with 80 g dietary protein/day showed that while there was a small decrease in absolute fat‐free mass, the relative fat‐free mass increased in the intervention group compared with the SoC group. Moreover, the amount of fat‐free mass lost did not differ from that observed in people without cirrhosis [26]. These results provide reassurance that weight loss is not detrimental to physical function in people with CC‐MASLD, while it improves overall body composition despite small reductions in absolute fat‐free mass. These changes were observed in conjunction with no worsening of physical performance using the comprehensive physical performance test and the liver frailty index, though most participants had very good baseline physical function, with 47% of them scoring the highest possible score in the test. Additionally, no intervention participant had a reduction in grip strength of at least 6.5 kg which has been considered as the minimal clinically meaningful reduction [27]. However, these results might not be generalisable to patients who are already at least moderately frail.

Cardiovascular disease is the second most common cause of death in CC‐MASLD after non‐hepatic cancers, therefore cardiovascular risk reduction is crucial in this population [28]. In our study, we found no evidence of significant between‐group changes in blood pressure or cholesterol. We observed a significant reduction in HbA1c levels at 16 weeks in the intervention group compared with the SoC group, but this was not maintained at 24 weeks. Irrespective of HbA1c improvement, the use of medications for type 2 diabetes and hypertension was reduced throughout the intervention, showing that the cardiometabolic risk profile did not worsen with less medication. Beyond cardiovascular risk management, people with CC‐MASLD have multiple co‐morbidities and might need listing for elective procedures, for which weight loss may be beneficial.

Although a definitive trial is required to assess whether low‐energy total diet replacement could improve clinically important outcomes, such as the incidence of hepatic decompensation or major cardiovascular endpoints, such a trial would necessitate a large multi‐ethnic cohort from multiple sites and a longer follow‐up period, given the relatively low frequency of decompensation events within a timeframe of 1–2 years. In the absence of licensed treatments, dietary energy restriction through a low‐energy total diet replacement programme appears to be a safe option to achieve significant weight loss and reduce liver fat without adverse effects in people with CC‐MASLD.

## Author Contributions

Concept: DAK, JFC, SAJ, PA, JWT. Design: DAK, JFC, SAJ, PA, JWT, MP, FS, FEM. Acquisition of data: DAK, JFC, FEM, ML, MP, FS. Analysis: DAK, FEM. Interpretation of data: All authors. Funding: SAJ, PA. Drafting of the manuscript: DAK. Critical revision and approval of the manuscript: All authors. Supervision: SAJ, JFC, JWT, PA.

## Conflicts of Interest

DAK, SAJ, JWT, MP, PA, and JFC are investigators in an investigator‐led trial publicly funded by the National Institute for Health and Care Research (NIHR), where the weight loss intervention was donated by Nestle Health Sciences and Oviva to the University of Oxford outside the submitted work (ISRCTN12900952). None of these associations led to payments to these authors. JWT has been part of the scientific advisory board for Novo Nordisk. MP is a shareholder in Perspectum, a University of Oxford spin out company, and has applied for a patent for medical imaging. No other conflicts of interest are reported.

## Data Availability Statement

Deidentified individual participant data are available from the corresponding author on reasonable request. All proposals requesting data access will need to complete a data request form with details of the research question and analysis plan.

Trial Steering and Data Monitoring Committee: Matthew Armstrong (chair), William Alazawi, Kelly Handley.

Guarantor of the Article: DAK

## Supporting information


**Table S1** Demographic and clinical characteristics at baseline.
**Figure S1** NAFLD activity score of each of the 15 participants with biopsy‐proven CCMASLD.
**Figure S2** Individual (dotted) and average (solid) absolute changes by group in AST, UKELD, and Child‐Pugh score.
**Table S2** Per‐protocol analysis.
**Table S3** Adverse events overview.
**Table S4** Alcohol intake (median [IQR]]) throughout the study based on 7‐day self‐reported intake at each time point.

Supporting information
