# Supplementary material for: Severe Dietary Energy Restriction for Compensated Cirrhosis Due to Metabolic Dysfunction‐Associated Steatotic Liver Disease: A Randomised Controlled Trial
Source: J Cachexia Sarcopenia Muscle. 2025 Apr 24;16(3):e13783. doi: 10.1002/jcsm.13783 (PMC12022231; doi:10.1002/jcsm.13783)
Supplement: Supplementary file 1 — Table S1 Demographic and clinical characteristics at baseline. Figure S1 NAFLD activity score of each of the 15 participants with biopsy‐proven CCMASLD. Figure S2 Individual (dotted) and average (solid) absolute changes by group in AST, UKELD, and Child‐Pugh score. Table S2 Per‐protocol analysis. Table S3 Adverse events overview. Table S4 Alcohol intake (median [IQR]]) throughout the study based on 7‐day self‐reported intake at each time point. [file JCSM-16-e13783-s001.pdf]

Supplement – Koutoukidis et al.

Procedures

Participants underwent magnetic resonance imaging and elastography (MRI and MRE) scans at baseline and 24 weeks, and vibration-controlled transient elastography scans at baseline, 16 weeks, and 24 weeks. The scans were conducted by trained operators and participants were fasted for at least 4 hours before the scans. MRI scans were performed with a Siemens Prisma 3T scanner (Siemens Healthineers, Erlangen, Germany). MRI-proton density fat fraction (PDFF) and T2* maps were determined using multiple-echo gradient recalled echo sequences (TR/TE_{min} = 15/1.1 ms, ΔTE = 1.1 ms, slice thickness 10 mm, FA = 3°, and bandwidth 1560 Hz/px). T1 maps were acquired using a standard shortened modified Look-Locker inversion recovery (shMOLLI) sequence. cT1 values were provided by Perspectum using the aforementioned sequences. The PDFF for two participants at baseline (36% each) was slightly above the usual upper threshold (≤35%) accepted for quantifying cT1. For these 2 scans, the measured cT1s were derived from 3 ROIs placed on a single slice (in contrast with the segmentation-based analysis performed for the rest of the data). Liver stiffness by MRE was determined using a spin echo sequence (4 slices, slice thickness 8 mm, TR/TE = 1000/47 ms, strong SPAIR fat suppression, matrix size 128 × 128, GRAPPA acceleration factor 2, bandwidth 2170 Hz/px, mechanical frequency 60 Hz). Liver stiffness maps were analysed as per the recommendations of the QIBA profile [1].

Blood samples were analysed using standard protocols in the hospital laboratory. Weight and fat-free mass were measured with bioelectrical impedance (TANITA SC-240MA, Tanita, Netherlands). After sitting for 5 minutes in a calm environment, blood pressure was measured 3 times with 1-minute intervals and the average of the last two measurements was used for analysis. Physical performance was measured with the objective modified physical performance test including 9 daily activities (e.g., performing a progressive Romberg test, climbing up and down four flights of 10 stairs). Each activity was scored 0-4

with the maximum score of 36 indicating high performance. Hand-grip strength was measured 3 times with a dynamometer (Takei 5401, Takei Scientific Instruments Co., Ltd, Tokyo, Japan) using the dominant hand. The grip strength measurements were combined with the chair stands, Romberg test, and sex, to calculate the Liver Frailty Index, for which higher scores indicate higher frailty [2]. Seven-day alcohol intake was assessed with an interview and heavy drinking was screened for with AUDIT-C. Participants filled in standardised demographic and feedback questionnaires. They also had a physical examination at baseline, 4, 16, and 24 weeks.

Medication adjustment guidance

TYPE 2 DIABETES		
**Patient currently takes:**	**Current dose**	**Recommendation**
Metformin		HALF daily dose
Liraglutide	0.6mg<br>1.2mg<br>1.8mg	STOP<br>REDUCE by 0.6mg<br>REDUCE by 0.6mg
Other Glucagon-like peptide-1 agonists		HALF daily dose
SGLT2 inhibitors		STOP
Sulphonylurea		STOP
Glitazone		STOP
Glinide		STOP
DPP IV inhibitor		STOP
Acarbose		STOP

At the end of the weight loss phase, re-assess patients' requirements for oral diabetic therapies using HbA1c measurements or a finger prick blood glucose measurement.

HYPERTENSION		
**Patient currently takes:**	**Current dose**	**Recommendation**
Loop Dieuretic:<br>*Furosemide*	≤ 40 mg daily<br>80 — 120 mg daily<br>≥ 120 mg daily	STOP<br>REDUCE by 40 mg daily<br>REDUCE by 40 mg daily
Bumetamide	≤ 1 mg daily<br>2-3 mg daily<br>≥ 3 mg daily	STOP<br>REDUCE to 1mg daily<br>REDUCE by 1mg daily
Thiazide Diuretic		STOP
α Blocker	Used for hypertension<br>Other uses	STOP<br>CONTINUE
ß Blocker		HALF daily dose
Ca channel blocker		HALF daily dose
ACE inhibitors or ARBs	Used for hypertension<br>Used for heart failure	STOP<br>HALF daily dose

LIPID-LOWERING DRUGS	
**Patient currently takes:**	**Recommendation**
Fibrates	STOP
Statins	CONTINUE
Ezetimibe	CONTINUE

Table S1: Demographic and clinical characteristics at baseline

Characteristic	Intervention	Standard of Care
Total, N	11	6
Age, years, median (IQR)	61.2 (7.5)	54.8 (7.5)
Sex, female	4 (36.4)	4 (66.7)
Ethnicity		
White	11 (100)	5 (83.3)
Asian	0 (0)	1 (16.7)
Education		
None	2 (18.2)	0 (0)
Up to secondary	2 (18.2)	2 (33.3)
Tertiary	6 (54.5)	4 (66.7)
Undisclosed	1 (9.1)	0 (0)
Annual household income		
<£15.5k	2 (18.2)	1 (16.7)
£15.5-25k	3 (27.3)	0 (0)
£26-39k	3 (27.3)	2 (33.3)
>=£40k	2 (18.2)	3 (50)
Undisclosed	1 (9.1)	0 (0)
Smoking status		
Never	4 (36.4)	2 (33.3)
Previous	6 (54.5)	3 (50)
Current	1 (9.1)	1 (16.7)
Type 2 diabetes	7 (63.6)	3 (50)
Hypertension	9 (81.8)	3 (50)
BMI $\geq$ 35 kg/m^2	5 (45.5)	5 (83.3)
BMI, kg/m^2, median (IQR)	34.9 (9)	37.5 (3.4)
AUDIT-C score, median (IQR)	2 (2.5)	2.5 (2.5)
Histological diagnosis	9 (81.8)	6 (100)
Time since diagnostic biopsy, months, median (IQR)	17.3 (28.5)	3.1 (28.5)
NAS score, median (IQR)	5 (2.5)	5 (2.5)

Figure S1: NAFLD activity score of each of the 15 participants with biopsy-proven CC-MASLD

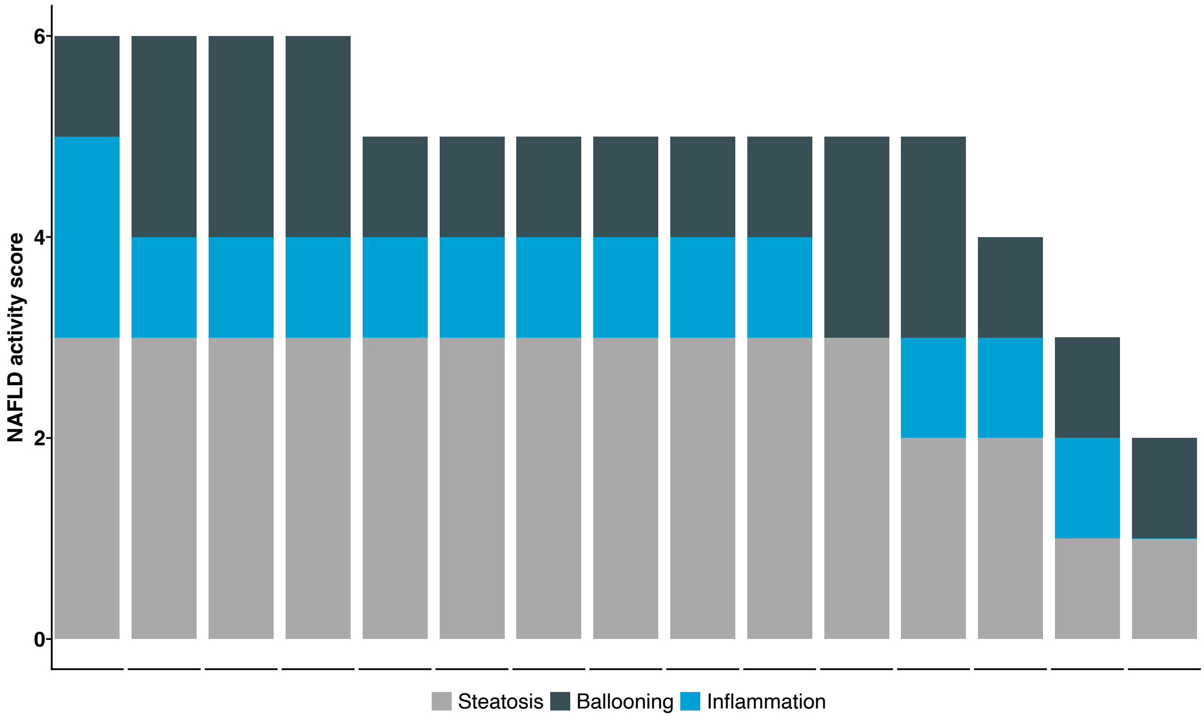

Figure S2: Individual (dotted) and average (solid) absolute changes by group in AST, UKELD, and Child-Pugh score.

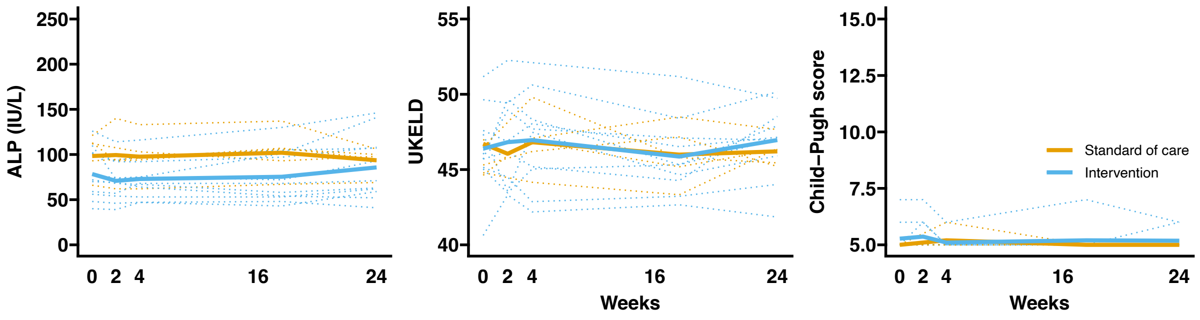

Table S2: Per-protocol analysis

Marker	Adjusted difference (95% CI)
Weight, kg	-14.5 (-19.5 to -9.5)
Fat-free mass, kg	-3.4 (-6.1 to -0.7)
Fat-free mass, %	6.9 (1.9 to 11.9)
Liver fat fraction, %	-7.5 (-11.7 to -3.3)
Controlled attenuation parameter, dB/m	-75 (-125.4 to -26.5)
corrected T1, ms	-153.4 (-273.8 to -33)
MRE-measured liver stiffness, kPa	0.5 (-1.2 to 2.2)
VCTE-measured liver stiffness, kPa	-2 (-8.4 to 4.7)
Enhanced liver fibrosis score	0.3 (-1.5 to 2.1)
ALT, IU/L	
AST, IU/L	
Bilirubin, µmol/L	
Conjugated bilirubin, µmol/L	
INR	
Prothrombin time	
Physical performance test	1.8 (-1.2 to 4.8)
Liver frailty index	1.6 (-8.7 to 11.8)
Grip strength, kg	0.2 (-0.4 to 0.8)
UKELD	0 (-1.8 to 1.9)
Child-Pugh score	-0.1 (-0.5 to 0.2)
Cholesterol to HDL ratio	-0.4 (-1.3 to 0.6)
Cholesterol, mmol/L	-0.2 (-1.2 to 0.8)
Haemoglobin A1c, %	-0.5 (-1.1 to 0.2)
Systolic blood pressure, mmHg	0.8 (-12.6 to 14.2)
Diastolic blood pressure, mmHg	-4.6 (-11.9 to 2.9)

Table S3: Adverse events overview

	Intervention, n(%)	Standard of care, n(%)
Participants with ≥1 adverse event	10 (91%)	4 (80%)
Adverse events reported by at least 2 participants in each group		
Headache	4 (36%)	0 (0%)
Constipation	3 (27%)	0 (0%)
COVID-19	3 (27%)	2 (40%)
Insomnia	2 (18%)	0 (0%)
Dizziness	2 (18%)	1 (20%)
Participants with adverse event grade ≥3	0 (0%)	0 (0%)
Adverse event leading to entering enhanced observation	0 (0%)	0 (0%)
Adverse event leading to discontinuation of the intervention	0 (0%)	N/A
N of serious adverse events	0 (0%)	0 (0%)
Pre-specified serious adverse events		
Hepatic decompensation	0 (0%)	0 (0%)
Liver transplantation	0 (0%)	0 (0%)
Hepatocellular carcinoma	0 (0%)	0 (0%)
Death	0 (0%)	0 (0%)

Table S4: Alcohol intake [median (IQR)] throughout the study based on 7-day self-reported intake at each time point.

	Intervention				Standard of care			
	Baseline	4 weeks	16 weeks	24 weeks	Baseline	4 weeks	16 weeks	24 weeks
**Alcohol units/week**	0 (0)	0 (0)	0 (0)	0 (0)	1 (5)	0 (4)	0 (4)	0 (0)

References

1. QIBA. Magnetic Resonance Elastography of the Liver. 2018 [11 July 2022]; Available from: https://qibawiki.rsna.org/images/a/a5/MRE-QIBAProfile-2018-05-02-CONSENSUS.pdf.
2. Wang CW, Lebsack A, Chau S, Lai JC. The Range and Reproducibility of the Liver Frailty Index. Liver Transpl. 2019;25(6):841-7. Epub 2019/03/19.
