# Supplementary material for: Severe Dietary Energy Restriction for Compensated Cirrhosis Due to Metabolic Dysfunction‐Associated Steatotic Liver Disease: A Randomised Controlled Trial
Source: J Cachexia Sarcopenia Muscle. 2025 Apr 24;16(3):e13783. doi: 10.1002/jcsm.13783 (PMC12022231; doi:10.1002/jcsm.13783)
Supplement: Supplementary file 2 — Supporting information [file JCSM-16-e13783-s002.pdf]

Study Title: Safety signals and potential efficacy of a low-energy total diet replacement programme with behavioural support to delay disease progression in compensated cirrhosis due to non-alcoholic fatty liver disease: a feasibility randomised controlled trial

Short title: LiFT 2: Low-energy treatment in compensated cirrhosis

Ethics Ref: 21/SC/0408

IRAS Project ID: 307043

Date and Version No: 12-May-23, 7.1

Chief Investigator: Dr Dimitrios Koutoukidis, Senior Research Fellow[1]

Investigators:
Prof Paul Aveyard, Professor of Behavioural Medicine[1]
Dr Jeremy F Cobbold, Consultant Hepatologist & Gastroenterologist[2]
Prof Jeremy W Tomlinson, Professor of Metabolic Endocrinology[3]
Dr Michael Pavlides, Head of Liver Imaging[4]
Prof Susan A Jebb, Professor of Diet and Population Health[1]
Dr Francesca Saffioti, Consultant Hepatologist & Gastroenterologist[2]

[1]Nuffield Dept. of Primary Care Health Sciences, University of Oxford
[2]Oxford University Hospitals NHS Foundation Trust
[3]Oxford Centre for Diabetes and Metabolism, University of Oxford
[4]Oxford Centre for Clinical Magnetic Resonance Research, University of Oxford

Sponsor: University of Oxford
Joint Research Office, Churchill Drive, Headington, Oxford OX3 7GB

Funder: National Institute for Health Research Oxford Biomedical Research Centre (NIHR Oxford BRC)

Chief Investigator Signature:

Statistician Signature:

Conflicts of interest: DK, PA, JFC, JWT, MP and SAJ report being investigators in a publicly-funded (NIHR) trial where the weight loss intervention was donated by Nestle Health Science to the University of Oxford outside the submitted work. MP is a shareholder for the company Perspectum Diagnostics, a University of Oxford spin out company, and has applied for a patent for medical imaging.

Confidentiality Statement

This document contains confidential information that must not be disclosed to anyone other than the Sponsor, the Investigator Team, HRA, host organisation, and members of the Research Ethics Committee, unless authorised to do so.

TABLE OF CONTENTS

1. KEY CONTACTS 6
2. LAY SUMMARY 7
3. SYNOPSIS 7
4. ABBREVIATIONS 9
5. BACKGROUND AND RATIONALE 11
6. OBJECTIVES AND OUTCOME MEASURES 13
7. STUDY DESIGN 14
8. PARTICIPANT IDENTIFICATION 14
8.1. Study Participants 15
8.2. Inclusion Criteria 15
8.3. Exclusion Criteria 15
9. PROTOCOL PROCEDURES 16
9.1. Recruitment 16
9.2. Informed Consent 17
9.3. Screening and Eligibility Assessment 17
9.4. Randomisation 18
9.5. Blinding and code-breaking 18
9.6. Description of study intervention, comparator, and study procedures (clinical) 18
9.6.1. Description of study intervention 18
9.6.2. Description of comparator 19
9.6.3. Description of study procedure(s) 19
9.7. Baseline Assessments 21
9.8. Subsequent Visits 21
9.9. Sample Handling 22
9.9.1 Sample handling for study purposes 22
9.10. Early Discontinuation/Withdrawal of Participants 22
9.11. Definition of End of Study 23
10. SAFETY REPORTING 23
10.1. Definition of Serious Adverse Events 24
10.2. Reporting Procedures for Serious Adverse Events 25
11. STATISTICS AND ANALYSIS 25
11.1. Statistical Analysis Plan (SAP) 25

11.2. Description of the Statistical Methods 25
11.3. Sample Size Determination 26
11.4. Analysis populations 26
11.5. Decision points 26
11.6. Stopping rules 26
11.7. The Level of Statistical Significance 26
11.8. Procedure for Accounting for Missing, Unused, and Spurious Data. 26
11.9. Procedures for Reporting any Deviation(s) from the Original Statistical Plan 26
11.10. Progression criteria to a full trial 27
12. DATA MANAGEMENT 27
12.1. Source Data 27
12.2. Access to Data 27
12.3. Data Recording and Record Keeping 28
12.4. Transfer of MRI Data to Perspectum Diagnostics, UK 29
13. QUALITY ASSURANCE PROCEDURES 29
13.1. Risk assessment 29
13.2. Study monitoring 29
13.3. Study Committees 29
14. PROTOCOL DEVIATIONS 30
15. SERIOUS BREACHES 30
16. ETHICAL AND REGULATORY CONSIDERATIONS 30
16.1. Declaration of Helsinki 30
16.2. Guidelines for Good Clinical Practice 30
16.3. Approvals 31
16.4. Other Ethical Considerations 31
16.5. Reporting 31
16.6. Transparency in Research 31
16.7. Participant Confidentiality 32
16.8. Expenses and Benefits 32
17. FINANCE AND INSURANCE 32
17.1. Funding 32
17.2. Insurance 32
17.3. Contractual arrangements 32
18. PUBLICATION POLICY 32

19. DEVELOPMENT OF A NEW PRODUCT/ PROCESS OR THE GENERATION OF INTELLECTUAL PROPERTY 32
19. ARCHIVING 32
20. REFERENCES 33
21. APPENDIX A: STUDY FLOW CHART 37
22. APPENDIX B: SCHEDULE OF STUDY PROCEDURES 38
APPENDIX C: STRUCTURE AND CONTENT OF THE INTERVENTION 40
23. APPENDIX D: ENHANCED OBSERVATION ALGORITHM 43
24. APPENDIX E: INTERVENTION DISCONTINUATION ALGORITHM 44
25. APPENDIX F: AMENDMENT HISTORY 45

1. KEY CONTACTS

**Chief Investigator**	Dr Dimitrios Koutoukidis<br><br>Nuffield Department of Primary Care Health Sciences, University of Oxford, Radcliffe Observatory Quarter, Woodstock Road, Oxford, OX2 6GG<br><br>T: +44 (0)1865 617767 E: dimitrios.koutoukidis@phc.ox.ac.uk
**Sponsor**	University of Oxford<br><br>RGEA, Joint Research Office, Boundary Brook House, Churchill Drive, Headington, Oxford OX3 7GB<br><br>T: +44 (0)1865 616480 E: RGEA.Sponsor@admin.ox.ac.uk
**Funder(s)**	National Institute for Health Research Oxford Biomedical Research<br><br>Centre (NIHR Oxford BRC)<br><br>Mary Logan<br><br>Nuffield Department of Primary Care Health Sciences, University of Oxford, Radcliffe Observatory Quarter, Woodstock Road, Oxford, OX2 6GG<br><br>E: mary.logan@phc.ox.ac.uk
**Clinical Trials Unit**	Not applicable
**Statistician**	Dr Dimitrios Koutoukidis<br><br>Nuffield Department of Primary Care Health Sciences, University of Oxford, Radcliffe Observatory Quarter, Woodstock Road, Oxford, OX2 6GG<br><br>T: +44 (0)1865 617767 E: dimitrios.koutoukidis@phc.ox.ac.uk
**Committees**	Trial Steering and Data Monitoring Committee Chair: Dr Matthew Armstrong, Consultant Hepatologist, University Hospitals Birmingham<br><br>Trial Management Committee Head: Dr Dimitrios Koutoukidis, CI<br><br>T: +44 (0)1865 617767 E: dimitrios.koutoukidis@phc.ox.ac.uk

2. LAY SUMMARY

The build-up of fat in the liver can cause inflammation and a lot of scarring in the liver. This condition is called compensated cirrhosis due to non-alcoholic fatty liver disease (CC-NAFLD). It is a serious disease that may require liver transplant and increases the risk of early death. It affects about 200,000 UK adults, most of whom are also living with obesity.

Currently, there is no drug available to treat CC-NAFLD. Weight loss programmes might improve it, but only if they lead to large weight loss. One programme that could achieve this on a large scale is a low-energy diet with professional support. In this programme, people eat only soups and shakes for 16 weeks (about 860 calories per day). Then, slowly over the next 8 weeks, they swap some soups and shakes for regular food. The support helps people stick to the programme and develop healthier eating habits. We know people lose weight rapidly and lower their risk of heart disease. This may also be a good treatment for CC-NAFLD, but there is a concern that rapid weight loss may worsen scarring in the liver. Here we will test the programme in a small group of people with detailed monitoring of the health of their liver to see if this concern is a reality.

We will recruit 24 adults with CC-NAFLD and excess weight. A computer will decide at random if patients continue with their care as usual (n=8) or are offered the programme (n=16). We will see whether enough patients are willing to take part in the trial, stick to the programme, and return for follow up visits. We will examine whether the treatment appears to be safe at 2, 4, 16, and 24 weeks with blood tests. If there are signs of worsening of the disease, we will stop the treatment. At the start of the programme and at 24 weeks, we will do a liver scan to make a more detailed assessment of liver scarring. We will also monitor changes in weight, body composition, risk of heart disease, and physical function.

This information will tell us if we can confidently plan a full trial to test whether this programme can delay progression of this liver disease.

We will publish the results in a scientific journal and present them to doctors treating liver disease.

3. SYNOPSIS

Study Title	Safety signals and potential efficacy of a low-energy total diet replacement programme with behavioural support to delay disease progression in compensated cirrhosis due to non-alcoholic fatty liver disease: a feasibility randomised controlled trial
Internal ref. no. / short title	LiFT 2: Low-energy treatment in compensated cirrhosis
Study registration	ISRCTN: https://www.isrctn.com/ISRCTN13053035
Sponsor	University of Oxford, RGEA<br>Joint Research Office, Churchill Drive, Headington, Oxford OX3 7GB
Funder	National Institute for Health Research Oxford Biomedical Research Centre (NIHR Oxford BRC)<br>E: mary.logan@phc.ox.ac.uk
Study Design	Randomised controlled trial

Study Participants	People with compensated cirrhosis due to non-alcoholic fatty liver disease and body mass index ≥ 30 kg/m^2		
Sample Size	24		
Planned Study Period	Total trial length: 3 years<br>Individual participant's involvement: approx. 6 months<br>Long-term follow-up via medical records: up to 2 years		
Planned Recruitment period	February 2022 – September 2022		
	Objectives	Outcome Measures	Timepoint(s)
Primary	i. To assess the safety signals of the intervention<br><br>ii. To assess the potential efficacy of the intervention in improving a marker of liver inflammation/fibrosis<br><br>iii. To assess the potential efficacy of the intervention in not worsening MRE-estimated liver stiffness, a marker of liver fibrosis	i. Criteria of severe changes in biochemistry (ALT, AST, total bilirubin)<br><br>ii. Iron-corrected T1 relaxation time (cT1) values by magnetic resonance imaging (MRI)<br><br>iii. Liver stiffness by magnetic resonance elastography (MRE)	0, 2, 4, 16, & 24<br><br>0 & 24<br><br>0 & 24
Secondary	To examine changes in liver stiffness by transient elastography<br><br>To examine changes in liver fat<br><br>To examine changes in blood biomarkers<br><br>To examine changes in physical performance<br><br>To examine adverse events<br><br>To examine changes in weight	Liver stiffness by transient elastography<br><br>i. Proton density fat fraction (PDFF)<br>ii. Controlled attenuation parameter<br><br>i. ELF<br>ii. UKELD score<br>iii. CP score<br><br>i. Physical performance test<br>ii. Liver frailty index<br><br>Adverse events<br><br>Body weight	0, 16, & 24<br><br>0 & 24<br>0, 16, & 24<br><br>0 & 24<br>0, 4, 16, & 24<br>0, 4, 16, & 24<br><br>0 & 24<br>0 & 24<br><br>0, 2, 4, 16, & 24<br><br>0, 4, 16, & 24

	To examine changes in total fat-free mass<br><br>To examine changes in visceral fat<br><br>To examine changes in muscle mass	Total fat-free mass on bioelectrical impedance<br><br>Visceral fat on MRI<br><br>Muscle mass on MRI	0, 4, 16, & 24<br><br>0 & 24<br><br>0 & 24
**Exploratory**	To examine changes in medication<br><br>To examine changes in cardiometabolic markers	Adjustment in the number and dose of medication<br><br>i. Blood pressure<br>ii. HbA1c<br>iii. Lipid profile	0, 4, 16, & 24<br><br>0, 4, 16, & 24<br>0, 16, & 24<br>0 & 24
**Process measures**	To examine feasibility of recruitment<br><br>To examine intervention engagement<br><br>To examine retention rate<br><br>To examine satisfaction with the intervention<br><br>To monitor alcohol intake throughout the study	i. Number of potentially eligible participants<br>ii. Proportion of eligible participants randomised<br>iii. Reasons for non-enrolment<br><br>i. Proportion of sessions attended<br>ii. Reasons for non-engagement<br><br>i. Proportion of randomised participants completing a 24-week follow-up visit<br>ii. Reasons for dropout<br><br>Feedback questionnaire<br><br>Alcohol intake questions	0<br>0<br>0<br><br>0, 4, 16, & 24<br><br>2, 4, 16, & 24<br><br>24<br><br>0, 4, 16, & 24
Intervention(s)	Low-energy total diet replacement programme with behavioural support.		
Comparator	Care as usual		

4. ABBREVIATIONS

ALP	Alkaline Phosphatase
ALT	Alanine Transaminase
AST	Aspartate Transaminase
AE	Adverse event
AUDIT-C	Alcohol Use Disorders Identification Test Consumption
BMI	Body Mass Index
CAP	Continuous Attenuation Parameter
CC-NAFLD	Compensated cirrhosis due to non-alcoholic fatty liver disease
CI	Chief Investigator
CP	Child-Pugh score
CRF	Case Report Form
cT1	Iron-corrected T1 relaxation time MRI sequence
ELF	Enhanced liver fibrosis test
GCP	Good Clinical Practice
GP	General Practitioner
HbA1c	Glycated haemoglobin
ICF	Informed Consent Form
INR	International normalized ratio
LUH	Liverpool University Hospitals NHS Foundation Trust
MHRA	Medicines and Healthcare products Regulatory Agency
MR	Magnetic Resonance
MRE	Magnetic Resonance Elastography
MRI	Magnetic Resonance Imaging
NAFLD	Non-alcoholic Fatty Liver Disease
NASH	Non-alcoholic Steatohepatitis
NHS	National Health Service
OUH	Oxford University Hospitals NHS Foundation Trust
PDFF	Proton density fat fraction
PHU	Portsmouth Hospitals University NHS Trust
PI	Principal Investigator
PT	Prothrombin time
R&D	NHS Trust R&D Department
REC	Research Ethics Committee
RCT	Randomised controlled trial

RGEA	Research Governance, Ethics & Assurance, University of Oxford
SAP	Statistical analysis plan
SOP	Standard Operating Procedure
TMF	Trial Master File
TDR	Low-energy total diet replacement programme with behavioural support
TSDMEC	Trial Steering, Data Monitoring, and Ethics Committee
U&E	Urea, creatinine, and electrolytes
UKELD	UK Model for end-stage liver disease

5. BACKGROUND AND RATIONALE

Compensated cirrhosis due to non-alcoholic fatty liver disease (CC-NAFLD) is a progressive and severe form of NAFLD. It can lead to decompensation and liver cancer requiring liver transplantation. It is estimated to affect 200,000 UK adults.[1 2] Compared with early-stage NAFLD and no fibrosis (stage 0), patients with compensated cirrhosis (i.e. fibrosis stage 4) due to NAFLD have lower quality of life and increased risk of liver-related and all-cause mortality by 42 and 6 times, respectively.[3-6] This costs the UK £363 million/year.[1] About 90% of patients have obesity. Obesity and weight gain are independently linked with disease progression.[7 8]

UK CC-NAFLD cases are projected to double to 400,000 in 2030.[2] This is estimated to increase decompensated cirrhosis and liver cancer by ~150%, increasing the need for liver transplantation.[9 10]

No pharmacotherapy to treat CC-NAFLD exists. In a James Lind Alliance priority setting partnership, finding effective treatments for NAFLD was the most important research question in the field.[11]

Our systematic reviews and meta-analyses of randomised controlled trials (RCTs) and pre-post trials of weight loss interventions showed that weight loss improves NAFLD and non-alcoholic steatohepatitis (NASH), a moderately severe form of NAFLD.[12 13] Data on fibrosis changes were limited and no study included patients with compensated cirrhosis. In patients with cirrhosis of any cause and obesity, a pilot weight loss trial with 5kg mean weight loss at 4 months raised no safety concerns and reduced hepatic portal hypertension.[14] In a longitudinal analysis of NASH and fibrosis stage 2-3 (n=215), the probability of histological “fibrosis improvement with no NASH worsening” after 1.5-2 years with 15% weight loss was 43% (95%CI: 26-62%), with no evidence that the association between weight loss and outcome depended upon baseline body mass index or fibrosis.[8] This indicated that moderate to advanced fibrosis, thought to be challenging to change, is modifiable with substantial weight loss.

Substantial weight loss through bariatric surgery, typically 20-35% at 1 year, improves biopsy-proven NASH in most patients and regresses fibrosis stage 2-3 in about half.[15] However, acceptability and access are limited. Less than 1% of all people eligible for bariatric surgery are treated in this way. A higher short-term risk of decompensation and mortality in cirrhosis also exists, likely due to the surgery itself than weight loss.[16]

Reductions in hepatocellular fat, injury, and inflammation (the NASH histological features) are seen as the drivers for fibrosis reduction among patients with NASH and fibrosis stage 2-3.[17] Therefore, substantial weight loss, by reducing these histological features, may delay progression to decompensation or even regress cirrhosis. Dietary approaches leading to substantial weight loss at scale would fill this gap but have not been tested and are not offered routinely.

One such approach is a low-energy total diet replacement programme with behavioural support (TDR). RCTs in routine care show that it leads to mean 15% weight loss (primarily fat mass loss) at 6 months, 10% weight loss at 1 year, 8% at 2 years, and 6% at 3 years.[18-21] It improves quality of life, and leads to clinically significant reductions in CVD risk and liver fat in early-stage NAFLD, and to type 2 diabetes (T2D) remission.[18 19 22] It is cost-effective with no evidence that it increases inequity.[23 24] NHS England is piloting a TDR programme in 5,000 people for T2D remission. However, active liver disease is excluded from the NHS roll-out. This follows the European Food Safety Authority advice on TDRs,[25] as safety concerns exist regarding liver worsening and frailty.

Rapid weight loss may temporarily increase liver fat and worsen liver disease.[26] A liver that is healthy or with early-stage NAFLD could handle a sharp metabolic change,[22 26] but this might not be the case in advanced liver disease. Early transient increases in alanine aminotransferase during TDR return to baseline levels by 1 month.[27] Liver biopsy data on TDRs are limited. A 400kcal/day TDR in 41 patients resulted in 34kg median weight loss for 4 months and showed that liver inflammation decreased in some but increased in others and 5 people that lost >1.6kg/week developed new fibrosis.[28] In 7 people with severe obesity on a 500kcal/day TDR for 5 months, liver fat improved, while other biopsy features did not change. After 1.5y, however, they had no evidence of NASH.[29] These data are somewhat reassuring that weight loss ultimately improves NASH but may suggest some early worsening, perhaps related to the rate of weight loss. Modern TDRs are less restrictive (860 kcal/day), which should reduce adverse events (AEs). Our ongoing pilot trial of TDR in patients with moderate fibrosis (but not cirrhosis) indicates improvements in liver fat, inflammation, and fibrosis based on magnetic resonance imaging (MRI) and magnetic resonance elastography (MRE) with no evidence of worsened fibrosis or inflammation assessed biochemically. Three cases with CC-NAFLD followed a 860 kcal/day TDR for 8 weeks in another recent pilot trial with no reported safety concerns.[30]

Weight loss in people with obesity and compensated cirrhosis remains controversial, because sarcopenic obesity is prevalent and weight loss, through muscle loss, may *theoretically* compromise physical function and increase frailty. However, despite the small reductions in muscle mass, substantial weight loss in frail but otherwise healthy older adults with obesity significantly improves physical function and reduces frailty.[31 32]

These uncertainties of the value of TDR to reduce fibrosis for people with CC-NAFLD require a definitive RCT to be resolved. Before a definitive trial, a small-scale RCT needs to examine whether people with CC-NAFLD can be recruited, stick to the treatment, and present for follow-up, whether the treatment appears to be safe, and to look for early signs of attenuation of the progression of this liver disease.

6. OBJECTIVES AND OUTCOME MEASURES

Objectives	Outcome Measures	Timepoint(s) in weeks of evaluation of this outcome measure (if applicable)
**Co-primary Objectives**		
i. To assess the safety signals of the intervention	i. Criteria of severe changes in biochemistry (ALT, AST, total bilirubin)	0, 2, 4, 16, & 24
ii. To assess the potential efficacy of the intervention in improving a marker of liver inflammation/fibrosis	ii. Iron-corrected T1 relaxation time (cT1) values by magnetic resonance imaging (MRI)	0 & 24
iii. To assess the potential efficacy of the intervention in not worsening MRE-estimated liver stiffness, a marker of fibrosis	iii. Liver stiffness by magnetic resonance elastography (MRE)	0 & 24
**Secondary Objectives**		
To examine changes in liver stiffness by transient elastography	Liver stiffness by transient elastography	0, 16, & 24
To examine changes in liver fat	i. Proton density fat fraction (PDFF)<br>ii. Controlled attenuation parameter	0 & 24<br>0, 16, & 24
To examine changes in blood biomarkers	i. ELF<br>ii. UKELD score<br>iii. CP score	0 & 24<br>0, 4, 16, & 24<br>0, 4, 16, & 24
To examine changes in physical performance	i. Physical performance test<br>ii. Liver frailty index	0 & 24<br>0 & 24
To examine adverse events	Adverse events	0, 2, 4, 16, & 24
To examine changes in weight	Body weight	0, 4, 16, & 24
To examine changes in total fat-free mass	Total fat-free mass on bioelectrical impedance	0, 4, 16, & 24
To examine changes in visceral fat	Visceral fat on MRI	0 & 24
To examine changes in muscle mass	Muscle mass on MRI	0 & 24
**Exploratory objectives**		
To examine changes in medication	Adjustment in the number and dose of medication	0, 4, 16, & 24

To examine changes in cardiometabolic markers	i. Blood pressure<br>ii. HbA1c<br>iii. Lipid profile	0, 4, 16, & 24<br>0, 16, & 24<br>0 & 24
**Process measures**		
To examine feasibility of recruitment	i. Number of potentially eligible participants<br>ii. Proportion of eligible participants randomised<br>iii. Reasons for non-enrolment	0<br>0<br>0
To examine intervention engagement	i. Proportion of sessions attended<br>ii. Reasons for non-engagement	0, 4, 16, & 24
To examine retention rate	i. Proportion of randomised participants completing a 24-week follow-up visit<br>ii. Reasons for dropout	2, 4, 16, & 24
To examine satisfaction with the intervention	Feedback questionnaire	24
To monitor alcohol intake throughout the study	Alcohol intake questions	0, 4, 16, & 24

7. STUDY DESIGN

This is a prospective randomised controlled trial single-blinded (outcome assessors) to provide indicative evidence of the safety signals and potential efficacy of a weight loss intervention in people with CC-NAFLD. Participants will be recruited from the Oxford University Hospitals NHS Foundation Trust (OUH), Liverpool University Hospitals NHS Foundation Trust (LUH), and Portsmouth Hospitals University NHS Trust (PHU). The intervention will be offered free of charge to participants through two private providers (Nestle Health Science and Oviva). Both providers are currently used within the NHS pilot scheme providing TDR for people with type 2 diabetes. Participants are expected to be involved in the study for approximately 6 months and will be requested to attend a screening visit, a baseline visit, 4 follow-up visits (at 2, 4, 16, and 24 weeks) for an appointment with a study clinician, a blood test, and an imaging scan (if applicable). These visits will take place in the respective local research sites and their collaborating University centres as applicable (e.g., for participants recruited from OUH, visits will be at the John Radcliffe Hospital that includes OUH and the University's Oxford Centre for Magnetic Resonance, and, for participants recruited from LUH, visits will be at LUH and the University of Liverpool). The intervention will be delivered over 24 weeks. Appendix A shows the flow-chart.

In designing this study, we have taken into account the opinions of people with NASH on the frequency of participant visits and the proposed tests. We also spoke to hepatologists around the country about their views on this programme.

8. PARTICIPANT IDENTIFICATION

8.1. Study Participants

Participants with compensated cirrhosis due to non-alcoholic fatty liver disease (CC-NAFLD), body mass index (BMI) ≥ 30 kg/m^2 and aged ≥18 years.

8.2. Inclusion Criteria

- Participant is able to communicate in English and is willing and able to give informed consent for participation in the trial.
- Aged ≥ 18 years.
- BMI ≥ 30 kg/m^2 (or BMI ≥ 27.5 kg/m^2 for people of Black, Asian, or minority ethnic origin as per the NICE guidance for obesity screening)[33]
- Diagnosed with CC-NAFLD based on one of the following
    - Biopsy with histological evidence of fibrosis score of 4 [with or without NASH (NASH defined as score of ≥1 for each of steatosis, inflammation, and ballooning] based on the NASH Clinical Research Network criteria
    - Previous biopsy with evidence of NASH but with current non-histological diagnosis of cirrhosis*
    - Previous biopsy or imaging with evidence of hepatic steatosis but with current non-histological diagnosis of cirrhosis*

  [*<u>*Definition of non-histological diagnosis of cirrhosis*</u>:

  Liver stiffness by transient elastography ≥15 kPA AND <u>ANY</u> of:

    - imaging evidence of nodular OR irregular liver AND/OR
    - presence of porto-systemic collateral vessels AND/OR
    - splenomegaly (without alternative cause) AND/OR
    - thrombocytopaenia in absence of primary haematological disease.]
- Stable dose of medication(s) for type 2 diabetes for at least 3 months prior to screening visit.
- Willing to allow his or her General Practitioner and consultant to be notified of participation in the trial.

8.3. Exclusion Criteria

The participant may not enter the trial if ANY of the following apply:

- Evidence for any of the following alternative or co-existing aetiologies: alcohol [alcohol screening tool (AUDIT-C) score ≥ 8, and for patients for whom alcohol may have been a contributing factor to their diagnosis of cirrhosis, they will be excluded if they have any history of sustained harmful alcohol intake defined as ≥35 units for females and ≥50 units for males per week], active viral hepatitis (subjects cured for hepatitis C virus infection less than 2 years prior to screening are not eligible), haemochromatosis, primary biliary cholangitis, primary sclerosing cholangitis, Wilson disease, severe alpha-1-antitrypsin deficiency (ZZ phenotype), and autoimmune hepatitis
- Alcohol intake of ≥ 18 units for females and ≥ 26 units for males over the last 7 days, as per the NAFLD diagnostic criteria.[34]
- Platelet count $<100\times10^9$ cells/L AND either medium (grade II) oesophageal or gastric varices with endoscopic high-risk stigmata (e.g., red signs), or large (grade III) varices on endoscopy within 1 year of screening [OR, IF NO ENDOSCOPY WITHIN 1 YEAR: exceeding the expanded Baveno VI criteria (platelet $< 110\times10^9$ cells/L AND/OR stiffness > 25 kPa)[35]]
- History or presence of hepatic decompensation (jaundice, ascites, hepatic encephalopathy, or variceal haemorrhage)

- Model for end-stage liver disease (MELD) score ≥ 13
- Child-Pugh score ≥ 8
- Total bilirubin > 25.5 µmol/L (Note: Patients with documented Gilbert's syndrome but conjugated bilirubin within normal range are eligible).
- ALT ≥ 5x upper limit of normal.
- AST ≥ 5x upper limit of normal.
- INR > 1.3
- HbA1c > 11.3% (>100mmol/mol).
- Insulin use for more than 10 years for type 2 diabetes management AND C-peptide <600pmol/L.
- Type 1 diabetes.
- Evidence of proliferative retinopathy.
- Listed for liver transplantation.
- History of hepatocellular carcinoma or history of hepatocellular carcinoma treatment.
- HIV infection
- Weight loss of 10% or more since diagnostic biopsy or, if biopsy not present, within the last 6 months.
- Previous bariatric surgery or ileal resection.
- History of biliary diversion.
- Acute cholecystitis or acute biliary obstruction.
- Contraindication to MRI.
- Documented arrhythmia, except atrial fibrillation, or prolonged QT syndrome.
- Taking warfarin.
- Chronic renal failure of stage 4 or 5.
- Scheduled for elective surgery under general anaesthesia
- Female participant who is pregnant, lactating, or planning pregnancy during the course of the trial.
- Any other significant disease or disorder which, in the opinion of the Investigator, may either put the participants at risk because of participation in the trial, or may influence the result of the trial, or the participant's ability to participate in the trial.
- Currently taking part in other interventional clinical trials unless approved by the CI.

9. PROTOCOL PROCEDURES

A schedule of procedures is available in Appendix B.

9.1. Recruitment

Participants will be recruited from OUH, LUH, and PHU. The Royal Berkshire Hospital NHS Foundation Trust will act as additional participant identification centre.

During the course of the patients' regular clinical appointment, clinicians will give those patients that are potentially eligible a participant information sheet. Following verbal consent, participants will be introduced to the research team for an eligibility assessment and a detailed discussion of the study at the end of their clinical appointment. The clinical team will also send invitation letters to potentially eligible participants. For participants who chose to contact the research team later or over the phone, the research team will explain the study and assess their eligibility over the phone.

In participant identification centres, clinicians will screen their clinic lists to identify potentially eligible participants. They will approach potentially eligible participants during the course of their routine appointment or send them letters inviting them to take part. They will check their medical history to see if they are likely to meet the inclusion and exclusion criteria and provide them with the participant information sheet. Participants who are interested to take part will be referred to one of the research sites (e.g., participants from Royal Berkshire NHS Foundation Trust will be referred to OUH) where the screening procedures and provision of informed consent will take place.

As part of the screening process, but after providing written informed consent, weight and height will be measured and a blood test will be performed at the hospital. Females may be asked to have a pregnancy test to confirm that they are not pregnant. They may also be advised to use an effective method of contraception for the duration of the trial, and we will provide them with information on suitable contraceptive methods, as necessary. Consented participants who are keen to proceed and who are potentially eligible will be booked for a baseline visit.

9.2. Informed Consent

The participant must personally sign and date the latest approved version of the Informed Consent form before any trial specific procedures are performed.

Written and verbal versions of the Participant Information and Informed Consent will be presented to the participants detailing no less than: the exact nature of the trial; what it will involve for the participant; the implications and constraints of the protocol; the known side effects and any risks involved in taking part. It will be clearly stated that the participant is free to withdraw from the trial at any time for any reason without prejudice to future care, without affecting their legal rights and with no obligation to give the reason for withdrawal.

The participant will be allowed as much time as wished to consider the information, and the opportunity to question the Investigator, their GP or other independent parties to decide whether they will participate in the trial. Written Informed Consent will then be obtained by means of participant dated signature and dated signature of the person who presented and obtained the Informed Consent. The person who obtained the consent must be suitably qualified and experienced, and have been authorised to do so by the Chief/Principal Investigator. A copy of the signed Informed Consent will be given to the participant and a copy will be filed in the medical notes. The original signed form will be retained at the trial site.

9.3. Screening and Eligibility Assessment

The screening procedures will initially involve checking the medical history and a brief interview to check concomitant medication, contraindications to MRI, and further eligibility criteria as listed in section 8. As per Appendix B, participants will be required to take a blood test and a measurement of weight and height. Their medical history and concomitant medication will be reviewed. They will also complete the 3-item Alcohol Use Disorders Identification Test consumption questionnaire (AUDIT-C).[36]

Following completion of the screening visit, the maximum duration between the screening visit and randomisation will be 4 weeks (28 days). After this period has passed and participants are still keen to participate, the screening blood test must be repeated to allow for an up-to-date measurement (e.g., during their baseline visit). In all cases, the screening blood test will need to occur within 28 days before randomisation.

Participants should also have their baseline assessment (section 9.6) during the window between screening visit and randomisation. If the baseline visit brings forth reasons to exclude the patient (e.g., participant is unable to undertake the MRI scan due to claustrophobia), the participant must not be

randomised; they will be regarded as screen failure, and will be replaced. Protocol waivers are not permitted.

Participants will not be randomised if a baseline visit has not been completed within 16 weeks from provision of informed consent. This cut-off will be in place to facilitate recruitment.

9.4. Randomisation

Consented participants will be individually randomised with a 2:1 allocation to receive either the intervention or care as usual through minimisation with a 20% random element.[37 38] The two stratified variables are BMI (≥/< 35 kg/m^2) and type 2 diabetes (yes/no), as these are prognostic factors of clinical outcomes.

Randomisation will occur after the baseline visit has been completed and a final check ensuring that all eligibility criteria have been met. To allow time for the analysis of the blood sample, the window between the end of the baseline visit and the randomisation will be within 24 hours.

The CI will programme the randomisation software (MinimPY). The software is validated and there will be no paper-based back-up randomisation procedure in case of emergencies. Upon completion of the baseline visit, the researcher carrying out the baseline visit will submit a request for randomisation via phone call to an independent researcher including the values of the stratification variables for the participant who completed the baseline visit. The independent researcher will run the minimisation software that will allocate the participant to one of the two groups and inform the CI of the group allocation over the phone. The CI will inform the participant of the group allocation over the phone, so that the participant does not need to return for a randomisation visit.

Allocation concealment[39] is achieved by

- concealing the processes of recruitment, screening, and eligibility assessment from the researcher performing the randomisation centrally
- performing the randomisation following the baseline visit
- not carrying out baseline visits for additional participants during the run-in period between the baseline visit of a participant and their randomisation.

9.5. Blinding and code-breaking

It is impossible to blind the participants and clinicians to the treatment due to the nature of the intervention. Therefore, procedures for breaking the allocation code are not applicable. The independent assessor of the MRI/MRE scans (for the assessment of the primary outcome) will be blinded to allocation.

9.6. Description of study intervention, comparator, and study procedures (clinical)

9.6.1. Description of study intervention

The intervention is a low-energy total diet replacement programme with behavioural support (TDR) and has three phases.

In phase 1 (sole source TDR, weeks 0-16), participants will consume a nutritionally complete package of 4 formula products per day [soups, shakes, and bars (860 kcal/day)].[40]

In phase 2 (food-re-introduction, weeks 17-22), products will be gradually reduced and replaced with food-based meals.[41] During this phase, participants will consume 3 products per day for weeks 17-18, 2

products per day for weeks 19-20, and 1 product per day for weeks 21-22 together with food-based meals.

In phase 3 (weight maintenance, weeks 23-24), participants will consume food-based meals.

Participants will have contact with the dietitian for around 15 minutes each week (or 30 minutes fortnightly) over the 24 weeks for behavioural support (for details on the structure and content of sessions please see Appendix C).[41] This will be over the phone or the app depending on participant preference.

The intervention including formula products and consultations will be donated by Nestle Health Science and Oviva, respectively (see section 17.1). Participants may choose to receive the formula products during their study assessments, by post, or a combination of both. Management of the stock of products (e.g. delivery, labelling, distribution, storage, and replacements) will follow a study-specific SOP.

Participants in the intervention group will continue to receive care as usual.

9.6.2. Description of comparator

Care as usual. Participants in the comparator control group will receive care as usual which includes, but not limited to, advice for healthy eating and weight loss by their doctor. This will allow for a comparison of the study intervention to the existing standard of care.

9.6.3. Description of study procedure(s)

<u>Demographic questionnaire (5 mins)</u>

Basic demographic characteristics, such as age, ethnicity, marital status, living arrangement, and smoking, will be captured using standardised questions.

<u>Blood sample collection (15 mins)</u>

About 10mL of blood will be drawn in each assessment. Samples will be sent to NHS labs for immediate analysis of ALT, AST, ALP, ELF, total and conjugated bilirubin, full blood count, INR, PT, U&E, and HbA1c.

<u>Weight and body composition (3 min)</u>

Weight will be measured with light clothing to the nearest 0.1kg using a calibrated digital scale which will also estimate body composition using bioelectrical impedance.

<u>Height (2 min)</u>

Height without shoes will be measured to the nearest 0.1cm using a stadiometer.

<u>Physical examination (15 min)</u>

Patients will have a physical examination. This may include but is not limited to an examination of the body of a participant for any possible signs or symptoms of medical conditions, such as examination for scleral icterus and pedal oedema, auscultation of the heart and lungs, and general physical findings (e.g., hepatosplenomegaly, peripheral manifestations of liver disease, ascites, wasting, or fetor). During the follow-up visits, this may also include discussion and examination to establish the presence of AEs participants might experience.

<u>Concomitant medication (5 min)</u>

Concomitant medication will be recorded.

<u>Blood pressure (10 mins)</u>

Blood pressure will be measured using a standardised protocol.[42] The participant will sit for 5 minutes before commencing the measurements. Three measurements will be taken at one-minute intervals. The mean of the last two measurements will be used for analysis.

Alcohol intake questions (5 mins)

Number of units of alcohol over the previous week will be estimated based on an interview on frequency and intensity of alcohol intake.

Objective physical performance test (20 mins)

Low scores in the modified physical performance test are associated with obesity and the test is responsive to weight loss interventions, with high test-retest reliability.[32] The total score is based on evaluation of nine daily activities. Each activity is scored between 0-4 following protocolised procedures; a total score of 36 indicates the highest performance.[43-45] The activities are

i. walking ~15 m
ii. putting on and removing a coat
iii. picking up a penny placed 30 cm in front of the participant's dominant foot
iv. standing up from a ~40cm chair
v. lifting a ~3 kg book from a table to a shelf ~ 30cm above shoulder height
vi. climbing one flight of 10 stairs
vii. performing a progressive Romberg test with feet side-by-side, semi-tandem, and full-tandem for a maximum of 10 seconds.
viii. climbing up and down four flights of 10 stairs
ix. performing a 360-degree turn.

Participants will also perform a hand-grip strength test in triplicate. Using their dominant hand, they will be instructed to squeeze with their maximum strength and hold steadily for a few seconds a standard hand-grip dynamometer. The grip strength measurements will be combined with the above chair stands, Romberg test, and sex to calculate the Liver Frailty Index.[46]

Transient elastography (15min)

Transient elastography (Fibroscan®) will be performed by a trained assessor to measure the continuous attenuation parameter (CAP) and liver stiffness. CAP and liver stiffness will provide an indication of liver steatosis and fibrosis, respectively. Participants will be assessed using the appropriate probe.

Liver MRI and MRE (approximately 45 mins of scanning time)

The study MR protocol does not require contrast or injected drugs. The reproducible iron-corrected T1 relaxation time (cT1) MRI sequence will provide information on liver inflammation and fibrosis by assessing the extracellular fluid component.[47] Liver fat will be quantified with proton density fat fraction (PDFF), which is an accurate, reproducible, and highly precise non-invasive biomarker of liver steatosis.[48]

Once contraindications to MRI are excluded by use of the facility's screening forms, the risks of undergoing a scan are minimal. A trained scanner operator or radiographer will go through a list of possible risks with the participant before scanning. The MRI scanner consists of a large powerful magnet. Magnetic resonance imaging uses no ionising radiation. There are, however, potential hazards associated with MRI and the scanning of participants including the presence of surgical implants, participants' clothing, jewellery (such as body piercings) bodily habitus, or medical conditions. A comprehensive list of potential risks has been compiled, and the participant will be checked against this by the operator, prior to entering the controlled areas of the MRI scanners. During the actual scanning procedure, the scanner produces loud banging noises and the participant will be given suitable hearing protection (earplugs) and the ear protection will be fitted correctly. During the experiment, the participant will be able to

communicate with the operator in the control room. In addition, they will be given a call button, which allows them to alert the operator at any time. People with a history of claustrophobia may be excluded from participation in the study. All participants will still be introduced carefully to the scanner and allowed to leave at any stage, should they wish to do so. Once in the scanner, participants will be able to indicate immediately if they wish the scanning to cease by pressing a call button in their hands.

The MRE scan will be performed immediately following the MRI scan, thereby extending the scanning time by approximately 15min. Where MRE will be performed, a plastic disc-like vibration source will be placed against the body wall over the region of the liver. This transmits low-frequency vibrations through the liver. This is generally well tolerated by patients and carries no additional risks or contraindications compared with MRI. The MRE scan will be performed only at sites that have the relevant MRE infrastructure.

<u>Feedback questionnaire (5 min)</u>

Participants will be given a study-specific version of a feedback questionnaire (adapted from a previous study[49]) on various aspects of the trial and of the intervention (for participants in the intervention group) to complete at home and return in a pre-paid envelope by post.

9.7. Baseline Assessments

The baseline assessment (week 0) will last approximately 2 hours and include the demographic questionnaire, blood sample (if screening blood sample >28 days old), weight and body composition, height, physical examination, concomitant medication, blood pressure, alcohol intake questions, objective physical performance test, transient elastography, and liver MRI and MRE (where applicable).

9.8. Subsequent Visits

The study schedule is available in Appendix B. The window periods will be:

- 7 days for the 2-week visit (3 days before the time the visit is expected to occur to 3 days after that)
- 2 weeks for the 4-week visit (one week before the time the visit is expected to occur to one week after that)
- 4 weeks for the 16-week visit (two weeks before the time the visit is expected to occur to two weeks after that)
- 6 weeks for the 24-week visit (three weeks before the time the visit is expected to occur to three weeks after that)

All efforts will be made for the participants to complete their visits within the above specified windows. In case of exceptional circumstances that may lead to participants having their assessments outside these window periods, we will run a sensitivity analysis to take this into account.

<u>Follow-up visit 1 at 2 weeks (± 3 days) (~30min)</u>

The first follow-up study visit will include a blood test and assessment of AEs (in line with section 10).

<u>Follow-up visit 2 at 4 weeks (± 1 week) (~1h)</u>

The second follow-up study visit will include the same assessments as the baseline visit with the addition of AEs but excluding the demographic questionnaire, height, transient elastography, liver MRI, MRE (where applicable), and HbA1c analysis.

<u>Follow-up visit 3 at 16 weeks (± 2 weeks) (~3h)</u>

The third follow-up study visit will include the same assessments as the baseline visit with the addition of AEs but with the exception of the demographic questionnaire and height.

Follow-up visit 4 at 24 weeks (± 3 weeks) (~3h)

The fourth follow-up study visit will include the same assessments as the follow-up visit 2 plus the feedback questionnaire.

Ad-hoc visits

Ad-hoc visits might take place based on the study clinician's judgement in case of an AE and in line with sections 9.9, 10, and 13.3.2.

Long-term follow-up through the hospital medical records

We will extract relevant data collected as part of routine care at OUH, LUH, and PHU as applicable for 2 years after each participant completes the study. The type and timing of relevant data collected will depend on data availability based on each participant's routine clinical care. This is likely to include weight, laboratory results, and health status.

9.9. Sample Handling

9.9.1 Sample handling for study purposes

A 10 ml (about 2 teaspoons) blood sample (~3ml for Barricor tube, ~4ml for EDTA tube, and ~2.5ml for sodium citrate tube) will be taken by a phlebotomist for examining eligibility (screening sample) and study outcomes (sample at baseline and follow-up 1-3 visits). Blood samples will be handled, analysed, and then disposed of locally at OUH, LUH, and PHU as applicable according to standard NHS procedures.

9.10. Early Discontinuation/Withdrawal of Participants

During the course of the study a participant may choose to withdraw early from the study treatment at any time. This may happen for several reasons, including but not limited to:

- The occurrence of what the participant perceives as an intolerable AE.
- Inability to comply with study procedures
- Participant decision

Participants in the care as usual group: They may opt out of study assessments but may remain on study follow-up.

Participants in the intervention group: They may stop the intervention and/or study assessments but may remain on study follow-up and will be encouraged to do so. However, if they choose not to attend the 2-week or 4-week safety assessment, they will not be able to continue the intervention but can remain on study follow-up. The visits should be completed within the specified window (as per section 9.8) but provisions will be made on a case-by-case basis to have this assessment completed outside this window in exceptional circumstances (e.g. if the patient is self-isolating due to COVID-19 but is keen to get assessed and resume the intervention).

All participants may also withdraw their consent, meaning that they wish to withdraw from the study completely. In the case of withdrawal from both treatment (intervention) and active follow up, the following options for a tiered withdrawal from the study will be given to participants and explained in the participant information sheet. According to the design of the trial, participants may have the following three options for withdrawal;

1) Participants may withdraw from active follow-up and further communication but allow the trial team to continue to access their medical records and any relevant hospital data that is recorded as part of routine standard of care; i.e., imaging scans, blood results, and disease progression data, etc.
2) Participants can withdraw from the study but permit data and samples obtained up until the point of withdrawal to be retained for use in the study analysis. No further data or samples would be collected after withdrawal.

In addition, the Investigator may discontinue a participant from the intervention at any time if the Investigator considers it necessary for any reason including, but not limited to:

- Pregnancy
- Ineligibility (either arising during the trial or retrospectively having been overlooked at screening)
- Significant protocol deviation
- Significant non-adherence with treatment regimen or trial requirements
- An adverse event which requires discontinuation of the trial intervention or results in inability to continue to comply with trial procedures
- Disease progression which requires discontinuation of the trial intervention or results in inability to continue to comply with trial procedures
- Criteria of the algorithm in Appendix E are met.
- If enhanced observation is necessary as per section 10 but is not possible

The follow-up of participants that have withdrawn from the intervention but not from active follow-up will continue with the standard follow-up assessments of the study.

If the intervention is discontinued due to criteria in Appendix E, the intervention may be re-introduced with approval of the Trial Steering, Data Monitoring, and Ethics Committee (TSDMEC) if another cause of the elevation is identified. If on re-introduction, the elevations in liver function tests persist, the intervention must be discontinued and enhanced observation must continue as per section 10. Data from randomised participants will be analysed and post-randomisation withdrawal from the study will not result in exclusion of the data for that participant from the analysis. If the participant is withdrawn before randomisation, they will be replaced (as per section 9.3). The type of withdrawal and reason for withdrawal will be recorded in the CRF. If the participant is withdrawn due to an AE, the Investigator will arrange for frequent telephone calls as agreed with the participant (or an ad-hoc study visit upon the study clinician's judgement) until the AE has resolved or stabilised (also see sections 9.7, 10, and 13.3.2). If a participant is withdrawn from treatment due to pregnancy the pregnancy will be followed-up to outcome. See the Safety Reporting section below.

9.11. Definition of End of Study

The end of trial is the point at which all the data has been entered and queries resolved.

10. SAFETY REPORTING

Potential AEs will be recorded at each follow-up assessment. Participants may contact the research team (e.g., over the phone) at any time point during the course of the study to report potential AEs. AEs will be recorded as part of the CRF of the subsequent study visit (e.g., an AE occurring between weeks 4 and 16 will be recorded as part of the 16-week assessment.

One previous trial reported no SAEs due to the TDR, whereas another reported two SAEs potentially related to the TDR in the same participant.[18 19] There was evidence that for every five people one would experience an adverse, mostly mild, event because of the TDR programme.[18] Constipation, headache, fatigue, and dizziness are the most common AEs albeit occurring in a minority of participants (<8% each) and disappeared over time.[18 19] Less common side effects include dry mouth, abdominal pain, bad breath, diarrhoea, hair loss, dry skin, mood changes, and feeling cold.

The safety reporting window begins from the first day of the intervention for participants in the intervention group and from the day of randomisation for participants in the control group. It finishes for both groups when the participant completes the study. The limit of investigator follow-up of SAEs will be until the participant completes the study. This requirement will be the same for all SAEs.

Laboratory abnormalities without clinical significance are not recorded as AEs or SAEs. However, laboratory abnormalities that require enhanced observation, medical or surgical intervention, or lead to discontinuation of the intervention must be recorded as an AE, as well as an SAE, if applicable.

Enhanced observation

Some patients with CC-NAFLD may have elevated liver function tests at baseline. Participant will enter into enhanced observation as per the algorithm in Appendix D. Enhanced observation will include:

- Repeating a blood test to assess ALT, ALP, total bilirubin, INR within 5 days of results.
- Obtaining a more detailed history of symptoms and prior or concurrent disease.
- Obtaining a history of concomitant drug use (including non-prescription medications and herbal and dietary supplement preparations), alcohol use, recreational drug use, adherence to the dietary intervention (including consumption of regular foods).
- Obtaining a history of exposure to environmental chemical agents.
- Ruling out other causes of liver disease as needed (obtain viral hepatitis panel, imaging for evaluation of biliary tract disease, etc, if required in the opinion of a medically qualified investigator).
- Continue to monitor the above tests weekly until the tests have fallen below the triggering threshold and then monthly for 2 months, and then as per protocol and until the end of the 24-week follow-up.

During enhanced observation, the intervention can be continued, if desired and results do not trigger intervention discontinuation (Appendix E), at the discretion of a medically qualified investigator.

Acute liver dysfunction

Due to the challenge of recognizing and diagnosing acute liver dysfunction in subjects with pre-existing liver dysfunction, the TSDMEC will review potential cases of acute liver dysfunction and classify them as:

- Acute liver dysfunction attributable to the intervention could be excluded (there is a clear alternative explanation)
- Acute liver dysfunction attributable to the intervention could not be excluded (there is no clear alternative explanation)
- Indeterminate due to insufficient data.

10.1. Definition of Serious Adverse Events

A serious adverse event is any untoward medical occurrence that:

- results in death
- is life-threatening
- requires inpatient hospitalisation or prolongation of existing hospitalisation
- results in persistent or significant disability/incapacity
- consists of a congenital anomaly or birth defect.

Other ‘important medical events’ may also be considered a serious adverse event when, based upon appropriate medical judgement, the event may jeopardise the participant and may require medical or surgical intervention to prevent one of the outcomes listed above.

NOTE: The term "life-threatening" in the definition of "serious" refers to an event in which the participant was at risk of death at the time of the event; it does not refer to an event which hypothetically might have caused death if it were more severe.

<u>Pre-specified clinical events judged as SAEs</u>
As one of the aims of the trial is to assess whether the intervention prevents progression of compensated cirrhosis to decompensation, the following events will be judged as SAEs:

- At least one event of hepatic decompensation:
  - Jaundice
  - Ascites
  - Hepatic encephalopathy
  - Variceal haemorrhage
- Liver transplantation or qualification for liver transplantation, defined as a MELD score ≥ 15
- Hepatocellular carcinoma.

10.2. Reporting Procedures for Serious Adverse Events

A serious adverse event (SAE) occurring to a participant will be reported to the REC that gave a favourable opinion of the study where in the opinion of the Chief Investigator the event was ‘related’ (resulted from administration of any of the research procedures) and ‘unexpected’ in relation to those procedures. Reports of related and unexpected SAEs will be submitted within 15 working days of the Chief Investigator becoming aware of the event, using the HRA report of serious adverse event form (see HRA website).

11. STATISTICS AND ANALYSIS

A Statistical Analysis Plan (SAP) is to be produced separately.

11.1. Statistical Analysis Plan (SAP)

The statistical aspects of the study are summarised here with details fully described in a statistical analysis plan that will be available from the time that the first participant is recruited. The SAP will be finalised before any analysis takes place.

11.2. Description of the Statistical Methods

A table will present the baseline characteristics. This table will include age, sex, ethnic group, weight, BMI, education, marital status, smoking status, health conditions, alcohol consumption, liver markers, and summary of medication. Continuous variables will be summarised using means and standard deviations. Medians with interquartile ranges will be presented where appropriate. Categorical variables will be summarised using counts and percentages. Data will be analysed using appropriate statistical software.

All randomised participants be included in the main analysis. Missing data will be imputed using appropriate methodology. Data will be plotted in before-after plots and visually inspected for changes in continuous outcomes. The percentage of participants with evidence of worsening in each liver outcome and the extent of this worsening will be reported.

Given the sample size, it is expected that the data will not be normally distributed and the statistical power to detect differences beyond the primary outcome will be limited.

Descriptive statistics ((mean [SD], median [IQR], count [%])) on each outcome, as appropriate, will be presented stratified by baseline cT1, Child-Pugh score, MELD score, diabetes status, and median BMI.

Where appropriate, continuous endpoints will be assessed with linear mixed effects models adjusting for treatment group and baseline value and binary endpoints will be assessed with the Cochran–Mantel–Haenszel test.

11.3. Sample Size Determination

An 88ms difference in cT1 (co-primary endpoint) is related with a 2-point change in the histological NAFLD activity score and a 1-point difference in histological ballooning (the main NASH feature).[50] In our ongoing pre-post pilot of TDR in NASH with fibrosis stage 2-3 (LiFT), cT1 reduced by a median of 130ms (SD of change: 70ms) at 12 weeks with similar estimates among the few participants who have already completed the 24-week follow-up.

Given the more advanced stage of the disease, 22 participants (n=15 intervention, n=7 control) will allow detecting a more conservative between-group difference of 100ms (SD: 70ms) with 90% power at 5% level. Assuming approximately a 10% dropout at 24 weeks as we observed in LiFT, 24 participants are needed (n=16 intervention, n=8 control).

11.4. Analysis populations

All randomised participants be included in the main analysis. A per protocol analysis will include the sub-sample of intervention participants who achieved ≥10.0% weight loss at 4 months. The adverse event analysis will include the participants commencing the intervention.

11.5. Decision points

No interim analysis is planned.

11.6. Stopping rules

The TSDMEC will formally recommend early termination if needed based on review of the AEs and SAEs. To facilitate TSDMEC decision making, the following recommendation for a pre-defined cut-off will apply. If no SAEs have occurred in the control group and the lower bound of the 95% confidence interval for SAEs is at least 30% in the intervention group based on a one-sample test for binomial proportion (Wilson score), the trial will stop.

11.7. The Level of Statistical Significance

The level of statistical significance will be set at $p<0.05$. There will be no adjustment for multiple testing.[51] The 95% confidence intervals will be presented but regarded nominal and descriptive.

11.8. Procedure for Accounting for Missing, Unused, and Spurious Data.

Missing data will be imputed using appropriate methodology as detailed in the SAP.

11.9. Procedures for Reporting any Deviation(s) from the Original Statistical Plan

Deviations from the original SAP will be reported and justified at publication following the CONSORT guidelines.

11.10. Progression criteria to a full trial

	Criterion<br>Decision	**Green**<br>**Progress**	**Amber**<br>**Progress with changes**	**Red**<br>**Stop**
1	Recruitment<br>(Proportion of eligible patients that have been randomised)	≥25%	11-24%	≤10%
2	Adherence<br>(Proportion of participants in the intervention group who commenced the programme with ≥10% weight loss at 16 weeks)	≥60%	46-59%	≤45%
3	Retention<br>(Proportion of randomised participants completing a 24-week follow-up visit)	≥85%	66-84%	≤65%
4	Safety profile	Based on adverse and serious adverse reactions. Adjudicated by the TSDMEC.		

12. DATA MANAGEMENT

The plan for the data management of the study are outlined below. There is not a separate Data Management document in use for the study.

12.1. Source Data

Source documents are where data are first recorded, and from which participants' CRF data are obtained. These include, but are not limited to, hospital records (from which medical history and previous and concurrent medication may be summarised into the CRF), clinical and office charts, laboratory and pharmacy records, radiographs, questionnaires, and correspondence.

CRF entries will be considered source data if the CRF is the site of the original recording (e.g. there is no other written or electronic record of data). All documents will be stored safely in confidential conditions. On all study-specific documents, other than the signed consent, the participant will be referred to by the study participant number/code, not by name.

12.2. Access to Data

Direct access will be granted to authorised representatives from the Sponsor and host institution for monitoring and/or audit of the study to ensure compliance with regulations.

The MRI scans will be processed by a third-party, Perspectum Diagnostics, using their software. See sections 12.3 and 17.3 for further details. The MRE scans will be processed within the University of Oxford.

Following participants' consent, Oviva will be granted access to the participants' name, home address, contact details, demographic details, and relevant medical information (such as weight, height, blood pressure, blood tests, medical history, and medication), so that the intervention sessions with the dietitians can take place. See section 12.3 for further details. Contractual arrangements will follow section 17.3.

Nestle Health Science will have no access to the study data.

De-identified data obtained as part of this study may be used in future research, here or abroad, and may involve commercial organisations.

12.3. Data Recording and Record Keeping

This study will be run using the Primary Care Clinical Trials Unit (CTU) standard operating procedures (SOPs) for guidance.

All trial data will be entered on to paper CRFs. Data will be transferred to the statistical software for analysis and validation checks will be performed (e.g., range checks). The participants will be identified by a unique trial specific number and/or code in any database. The name and any other identifying detail will NOT be included in any trial data electronic file.

The Oviva electronic medical record and the Oviva app are a single technology platform called Oviva Coaching Suite which is compliant with the NHS Digital Data Security and Protection Toolkit. Oviva will retain the participants' data for up to 8 years in line with the NHS Digital Guidance. Oviva will transfer the personal information for secure storage to Switzerland where the required information security standards are being followed. The Oviva-related data management will be in line with the University's Information Security and Data Protection Impact Assessment.

We will only share the following de-identified research data with Perspectum Diagnostics: MRI/MRE scan, age, sex, weight, height, blood test results, and ultrasound results. We will not share any personally identifiable information. Perspectum Diagnostics will retain the data for a maximum of 5 years and may utilise them for future internal research projects in line with the existing Collaboration Agreement with the University of Oxford. Further details of the data sharing between the two parties and relevant data analysis are pre-specified in the Collaboration Agreement.

We will store the de-identified research data (e.g. lab results, questionnaire data) indefinitely and any research documents with personal information (i.e., consent forms) for 3 years securely at the University of Oxford after the end of the study. Hard copies of personal data will be stored in a locked cupboard in a room with limited access at the University of Oxford.

The research and clinical teams will use participant contact details to contact individuals about the research study and make sure that relevant information about the study is recorded for participant care, and to oversee the quality of the study. We will keep identifiable information about participants (i.e., contact details) from this study separate from the research data and for up to 1 year after the study has finished, so that we can inform participants of the results of the study. We will only keep bank details of each participant who provide them for reimbursement of expenses for 7 years after study completion as per University policy.

Prior to database lock, a dataset review will be undertaken by the CI to ensure all queries have been resolved and the dataset is complete.

The Data Management will be compliant with the relevant Sponsor organisation's policy.

12.4. Transfer of MRI Data to Perspectum Diagnostics, UK

Perspectum Diagnostics provides a dedicated, secure cloud-based portal called quantitative analysis services (QAS) for data and study management (ISO 270001, 21CFR11). This cloud-based image analysis system is hosted in the United States. The portal provides a complete end-to-end solution for anonymised imaging data upload, archiving, monitoring and return as well as troubleshooting queries and automated data alerts. There will be no identifiable information uploaded via this portal. All MRI data will already be de-identified at the time of image acquisition. Participants will be identified in the Perspectum Diagnostic portal by only a second participant ID number. Specifically, a second participant ID unique to the baseline or 24-week scan will be generated which will consist of a random number between 0 and 300, which will be different from the original study ID number, and will be linked with the original study ID. The key between the two study ID numbers will be held securely at the University of Oxford by the CI. At the end of the study, this key which links the two study IDs will be securely destroyed. This process serves two purposes: it does not allow the scan to be identified as baseline or follow-up scan on the system enhancing the blinding process and aims to minimise the risk that the data held by Perspectum Diagnostics can be linked back to the rest of the data held by the University.

Linked-anonymised image DICOM files will be exported securely (encrypted and password protected) using the portal to Perspectum Diagnostics. Data protection would be our paramount importance. Data will not leave the OCMR system until all patient identifiable details are removed. Although data is de-identified, it will still be treated with the same level of security as containing patient details. The de-identified images will be analysed at a dedicated workstation at OCMR. There is a research collaboration agreement between University of Oxford and Perspectum Diagnostics dated 4th September 2014. De-identified data that has been sent to Perspectum Diagnostics may be used for their internal research and development purposes. It will be governed by the amendments made to section 11 of the existing research collaborative agreement between University of Oxford and Perspectum Diagnostics. Hard copy of MRI data files will be stored securely at the study site (OCMR).

13. QUALITY ASSURANCE PROCEDURES

The study may be monitored, or audited in accordance with the current approved protocol, GCP, relevant regulations and standard operating procedures.

13.1. Risk assessment

A risk assessment and monitoring plan will be prepared before the study opens and will be reviewed as necessary over the course of the study to reflect significant changes to the protocol or outcomes of monitoring activities.

13.2. Study monitoring

Regular monitoring will be performed according to the current PC-CTU procedures by the study team. Data will be evaluated for compliance with the protocol and accuracy in relation to source documents. Following written SOPs, the monitors will verify that the clinical study is conducted and data are generated, documented and reported in compliance with the protocol, GCP and the applicable regulatory requirements. The procedure outlined in 13.3.2 will be followed for the data that require expert analysis and/or interpretation.

13.3. Study Committees

13.3.1 Study Management Group

The study management group will comprise of all named investigators and the trial manager and it will be responsible for the day-to-day running of the trial. The trial manager will update via email the group monthly on trial progress. The group will meet quarterly to evaluate progress and the frequency of the meetings will be adjusted depending on progress.

13.3.2 Trial Steering, Data Monitoring, and Ethics Committee (TSDMEC)

As this is an unblinded trial, the Trial Steering Committee and the Data Monitoring and Ethics Committee will be combined. The TSDMEC of 3 independent members [chair (hepatologist), academic with expertise in clinical trials, clinician] will review and meet online to approve the protocol and meet thereafter every 6 months or following completion of the 4-week follow-up of 8 randomised participants (whichever is earlier) to review data quality and monitor ethics and safety based on unblinded data and advise the TMG. The TSDMEC may meet more frequently and request to review specific analyses (e.g. comparing rates of AEs by trial group or other subgroup) at their discretion and as needed. They will review an extra safety profile report between the scheduled meetings of biochemistry and relevant data of participants discontinuing the intervention or being on enhanced observation. They will review SAEs as they occur. They will formally recommend at the end of each meeting whether the trial should continue with no changes, continue with protocol modifications, or be stopped. Its role, constitution, and composition will follow NIHR guidelines.

14. PROTOCOL DEVIATIONS

A study-related deviation is a departure from the ethically approved study protocol or other study document or process (e.g., consent process or administration of study intervention) or from Good Clinical Practice (GCP) or any applicable regulatory requirements. Any deviations from the protocol will be documented in a protocol deviation form and filed in the study master file. The PC-CTU SOPs will be followed for identifying non-compliances, escalation to the central team and assessment of whether a non-compliance /deviation may be a potential Serious Breach.

15. SERIOUS BREACHES

A “serious breach” is a breach of the protocol or of the conditions or principles of Good Clinical Practice which is likely to affect to a significant degree –

(a) the safety or physical or mental integrity of the trial subjects; or

(b) the scientific value of the research.

In the event that a serious breach is suspected the Sponsor will be contacted within 1 working day. In collaboration with the CI, the serious breach will be reviewed by the Sponsor and, if appropriate, the Sponsor will report it to the approving REC committee and the relevant NHS host organisation within seven calendar days.

16. ETHICAL AND REGULATORY CONSIDERATIONS

16.1. Declaration of Helsinki

The Investigator will ensure that this study is conducted in accordance with the principles of the Declaration of Helsinki.

16.2. Guidelines for Good Clinical Practice

The Investigator will ensure that this study is conducted in accordance with relevant regulations and with Good Clinical Practice.

16.3. Approvals

Following Sponsor approval, the protocol, informed consent form, and participant information sheet will be submitted to an appropriate Research Ethics Committee (REC), and HRA (where required) and host institutions for written approval.

The Investigator will submit and, where necessary, obtain approval from the above parties for all substantial amendments to the original approved documents.

16.4. Other Ethical Considerations

Observational data show that NASH resolves and early-stage fibrosis improves at one year in 90% and 45%, respectively, of patients with at least 10% weight loss at one year. The intervention under examination here has been previously shown to achieve this weight loss in almost half of the participants with rapid weight loss at the early phase. Previous studies that have given rise to the belief that rapid weight loss may worsen liver inflammation and fibrosis have provided extreme energy deficits and, in one case, providing hospitalised participants with no food at all. Our aim is to observe whether this occurs with more modest energy restriction in patients with CC-NAFLD. However, we observed no such concerns in our ongoing LiFT trial in patients with moderately severe liver disease (NASH and moderate fibrosis). We will follow-up participants with a detailed investigation at 4 weeks, 16 weeks, and 24 weeks to check for changes in their liver disease using blood tests as well as MR scans at 24 weeks.

In the unlikely event of seeing any structural abnormalities on a scan or blood test, these will be checked by a clinical specialist. If the abnormality is deemed medically important, the research team will refer the participant to their clinical care team who will discuss the implications with the participant and arrange for further investigations as necessary. Participants will not be informed unless the doctor considers the finding has clear implications for their current or future health. It is important to note that scans are not carried out for diagnostic purposes, and therefore the scans are not a substitute for a clinical appointment. Rather, the scans are intended for research purposes only.

AEs during the intervention period have previously shown to be infrequent and disappear over time (see section 10). The dietitian will support the participants based on current clinical guidance during the consultations in managing potential AEs. The research team will handle the AEs when reported in a sensitive manner and will communicate these to the hepatology clinical team in line with section 9.7, 9.9, 10, and 13.3.2.

16.5. Reporting

The CI shall submit once a year throughout the study, or on request, an Annual Progress report to the REC Committee, HRA (where required) host organisation, Sponsor and funder (where required). In addition, an End of Study notification and final report will be submitted to the same parties.

16.6. Transparency in Research

Prior to the recruitment of the first participant, the trial will have been registered on a publicly accessible database.

Where the trial has been registered on multiple public platforms, the trial information will be kept up to date during the trial, and the CI or their delegate will upload results to all those public registries within 12 months of the end of the trial declaration.

16.7. Participant Confidentiality

The study will comply with the UK General Data Protection Regulation (GDPR) and Data Protection Act 2018, which require data to be de-identified as soon as it is practical to do so. The processing of the personal data of participants will be minimised by making use of a unique participant study number only on all study documents and any electronic database(s), with the exception of the CRF, where participant initials may be added. All documents will be stored securely and only accessible by study staff and authorised personnel. The study staff will safeguard the privacy of participants' personal data.

16.8. Expenses and Benefits

Reasonable travel expenses up to £30 for any visits additional to normal care will be reimbursed on production of receipts, or a mileage allowance provided as appropriate.

17. FINANCE AND INSURANCE

17.1. Funding

This study is funded by the NIHR Oxford Biomedical Research Centre (BRC). Nestle Health Science and Oviva will provide the intervention, i.e., the formula products and the dietitians' time, respectively. Perspectum Diagnostics will provide the MRI analysis.

17.2. Insurance

The University has a specialist insurance policy in place which would operate in the event of any participant suffering harm as a result of their involvement in the research (Newline Underwriting Management Ltd, at Lloyd's of London). NHS indemnity operates in respect of the clinical treatment that is provided.

17.3. Contractual arrangements

Appropriate contractual arrangements will be put in place with all third parties.

18. PUBLICATION POLICY

The Investigators will be involved in reviewing drafts of the manuscripts, abstracts, press releases, and any other publications arising from the study. Authors will acknowledge that the study was funded by the NIHR Oxford BRC, that the intervention was be provided by Nestle Health Science and Oviva, and that the MRI analysis was undertaken by Perspectum Diagnostics. None of them will have any role in the study design; collection, management, statistical analysis, and interpretation of data; writing of the reports; and the decision to submit the reports for publication. Authorship will be determined in accordance with the ICMJE guidelines and other contributors will be acknowledged.

We will send a lay summary of the trial results to participants at the end of the study.

19. DEVELOPMENT OF A NEW PRODUCT/ PROCESS OR THE GENERATION OF INTELLECTUAL PROPERTY

Ownership of IP generated by employees of the University vests in the University. The University will ensure appropriate arrangements are in place as regards any new IP arising from the trial.

19. ARCHIVING

On completion of the trial and data cleaning, the study documentation, including participant identifiable information, will be transferred to a secure, GCP compliant archiving facility, where they will be held and archived. Please see section 12.3 for the length of archiving of each type of data. The database will be anonymised and a secure compact disc containing the link between identification number and participant identifiable information will be stored in a secure archiving facility.

20. REFERENCES

1. Younossi ZM, Blissett D, Blissett R, et al. The economic and clinical burden of nonalcoholic fatty liver disease in the United States and Europe. *Hepatology* 2016;64(5):1577-86. doi: 10.1002/hep.28785 [published Online First: 2016/10/22]
2. Estes C, Anstee QM, Arias-Loste MT, et al. Modeling NAFLD disease burden in China, France, Germany, Italy, Japan, Spain, United Kingdom, and United States for the period 2016-2030. *J Hepatol* 2018;69(4):896-904. doi: 10.1016/j.jhep.2018.05.036 [published Online First: 2018/06/11]
3. Kennedy-Martin T, Bae JP, Paczkowski R, et al. Health-related quality of life burden of nonalcoholic steatohepatitis: a robust pragmatic literature review. *J Patient Rep Outcomes* 2017;2:28. doi: 10.1186/s41687-018-0052-7 [published Online First: 2018/07/10]
4. Ekstedt M, Hagstrom H, Nasr P, et al. Fibrosis stage is the strongest predictor for disease-specific mortality in NAFLD after up to 33 years of follow-up. *Hepatology* 2015;61(5):1547-54. doi: 10.1002/hep.27368 [published Online First: 2014/08/16]
5. Angulo P, Kleiner DE, Dam-Larsen S, et al. Liver Fibrosis, but No Other Histologic Features, Is Associated With Long-term Outcomes of Patients With Nonalcoholic Fatty Liver Disease. *Gastroenterology* 2015;149(2):389-97 e10. doi: 10.1053/j.gastro.2015.04.043 [published Online First: 2015/05/04]
6. Dulai PS, Singh S, Patel J, et al. Increased risk of mortality by fibrosis stage in nonalcoholic fatty liver disease: Systematic review and meta-analysis. *Hepatology* 2017;65(5):1557-65. doi: 10.1002/hep.29085 [published Online First: 2017/01/29]
7. Berzigotti A, Garcia-Tsao G, Bosch J, et al. Obesity is an independent risk factor for clinical decompensation in patients with cirrhosis. *Hepatology* 2011;54(2):555-61. doi: 10.1002/hep.24418 [published Online First: 2011/05/14]
8. Koutoukidis DA, Jebb SA, Tomlinson JW, et al. Association of weight changes with changes in histological features and blood markers in non-alcoholic steatohepatitis. *Clin Gastroenterol Hepatol* 2021 doi: 10.1016/j.cgh.2021.03.047 [published Online First: 2021/04/05]
9. Estes C, Razavi H, Loomba R, et al. Modeling the epidemic of nonalcoholic fatty liver disease demonstrates an exponential increase in burden of disease. *Hepatology* 2018;67(1):123-33. doi: 10.1002/hep.29466 [published Online First: 2017/08/13]
10. Noureddin M, Vipani A, Bresee C, et al. NASH Leading Cause of Liver Transplant in Women: Updated Analysis of Indications For Liver Transplant and Ethnic and Gender Variances. *Am J Gastroenterol* 2018;113(11):1649-59. doi: 10.1038/s41395-018-0088-6 [published Online First: 2018/06/09]
11. Gurusamy KS, Walmsley M, Davidson BR, et al. Top research priorities in liver and gallbladder disorders in the UK. *BMJ Open* 2019;9(3):e025045. doi: 10.1136/bmjopen-2018-025045 [published Online First: 2019/03/10]
12. Koutoukidis DA, Astbury NM, Tudor KE, et al. Association of Weight Loss Interventions With Changes in Biomarkers of Nonalcoholic Fatty Liver Disease: A Systematic Review and Meta-analysis. *JAMA Intern Med* 2019;179(9):1262-71. doi: 10.1001/jamainternmed.2019.2248 [published Online First: 2019/07/02]

13. Koutoukidis DA, Koshiaris C, Henry JA, et al. The effect of the magnitude of weight loss on non-alcoholic fatty liver disease: a systematic review and meta-analysis. *Metabolism* 2021;115:154455. doi: 10.1016/j.metabol.2020.154455
14. Berzigotti A, Albillos A, Villanueva C, et al. Effects of an intensive lifestyle intervention program on portal hypertension in patients with cirrhosis and obesity: The SportDiet study. *Hepatology* 2017;65(4):1293-305. doi: 10.1002/hep.28992 [published Online First: 2016/12/21]
15. Fakhry TK, Mhaskar R, Schwitalla T, et al. Bariatric surgery improves nonalcoholic fatty liver disease: a contemporary systematic review and meta-analysis. *Surg Obes Relat Dis* 2019;15(3):502-11. doi: 10.1016/j.soard.2018.12.002 [published Online First: 2019/01/27]
16. Ahmed S, Pouwels S, Parmar C, et al. Outcomes of Bariatric Surgery in Patients with Liver Cirrhosis: a Systematic Review. *Obes Surg* 2021 doi: 10.1007/s11695-021-05289-x [published Online First: 2021/02/18]
17. Brunt EM, Kleiner DE, Wilson LA, et al. Improvements in Histologic Features and Diagnosis Associated With Improvement in Fibrosis in Nonalcoholic Steatohepatitis: Results From the Nonalcoholic Steatohepatitis Clinical Research Network Treatment Trials. *Hepatology* 2019;70(2):522-31. doi: 10.1002/hep.30418 [published Online First: 2018/12/15]
18. Astbury NM, Aveyard P, Nickless A, et al. Doctor Referral of Overweight People to Low Energy total diet replacement Treatment (DROPLET): pragmatic randomised controlled trial. *BMJ* 2018;362:k3760. doi: 10.1136/bmj.k3760 [published Online First: 2018/09/28]
19. Lean ME, Leslie WS, Barnes AC, et al. Primary care-led weight management for remission of type 2 diabetes (DiRECT): an open-label, cluster-randomised trial. *Lancet* 2018;391(10120):541-51. doi: 10.1016/S0140-6736(17)33102-1 [published Online First: 2017/12/10]
20. Lean MEJ, Leslie WS, Barnes AC, et al. Durability of a primary care-led weight-management intervention for remission of type 2 diabetes: 2-year results of the DiRECT open-label, cluster-randomised trial. *Lancet Diabetes Endocrinol* 2019;7(5):344-55. doi: 10.1016/S2213-8587(19)30068-3 [published Online First: 2019/03/11]
21. Astbury N, Edwards R, Ghebretinsea F, et al. Extended follow-up of a short total diet replacement programme: results of the Doctor Referral of Overweight People to Low Energy total diet replacement Treatment (DROPLET) randomised controlled trial at 3 years. Under review
22. Taylor R, Al-Mrabeh A, Zhyzhneuskaya S, et al. Remission of Human Type 2 Diabetes Requires Decrease in Liver and Pancreas Fat Content but Is Dependent upon Capacity for beta Cell Recovery. *Cell Metab* 2018;28(4):547-56 e3. doi: 10.1016/j.cmet.2018.07.003 [published Online First: 2018/08/07]
23. Kent S, Aveyard P, Astbury N, et al. Is Doctor Referral to a Low-Energy Total Diet Replacement Program Cost-Effective for the Routine Treatment of Obesity? *Obesity (Silver Spring)* 2019;27(3):391-98. doi: 10.1002/oby.22407 [published Online First: 2019/02/26]
24. Astbury NM, Tudor K, Aveyard P, et al. Heterogeneity in the uptake, attendance, and outcomes in a clinical trial of a total diet replacement weight loss programme. *BMC Med* 2020;18(1):86. doi: 10.1186/s12916-020-01547-4 [published Online First: 2020/04/17]
25. EFSA Panel on Dietetic Products NaAN. Scientific Opinion on the essential composition of total diet replacements for weight control. *EFSA Journal* 2015;13:3957.
26. Rolland C, Mavroeidi A, Johnston KL, et al. The effect of very low-calorie diets on renal and hepatic outcomes: a systematic review. *Diabetes Metab Syndr Obes* 2013;6:393-401. doi: 10.2147/DMSO.S51151 [published Online First: 2013/10/22]
27. Jhaveri MA, Anderson JW. Sequential changes of serum aminotransferase levels in severely obese patients after losing weight through enrollment in a behavioral weight loss program. *Postgrad Med* 2010;122(4):206-12. doi: 10.3810/pgm.2010.07.2188 [published Online First: 2010/08/03]
28. Andersen T, Gluud C, Franzmann MB, et al. Hepatic effects of dietary weight loss in morbidly obese subjects. *J Hepatol* 1991;12(2):224-9. [published Online First: 1991/03/01]
29. Drenick EJ, Simmons F, Murphy JF. Effect on hepatic morphology of treatment of obesity by fasting, reducing diets and small-bowel bypass. *N Engl J Med* 1970;282(15):829-34. doi: 10.1056/NEJM197004092821502 [published Online First: 1970/04/09]

30. Scragg J, Avery L, Cassidy S, et al. Feasibility of a Very Low Calorie Diet to Achieve a Sustainable 10% Weight Loss in Patients With Nonalcoholic Fatty Liver Disease. *Clinical and Translational Gastroenterology* 2020;11(9):e00231. doi: 10.14309/ctg.0000000000000231
31. Villareal DT, Aguirre L, Gurney AB, et al. Aerobic or Resistance Exercise, or Both, in Dieting Obese Older Adults. *N Engl J Med* 2017;376(20):1943-55. doi: 10.1056/NEJMoa1616338 [published Online First: 2017/05/18]
32. Villareal DT, Chode S, Parimi N, et al. Weight loss, exercise, or both and physical function in obese older adults. *N Engl J Med* 2011;364(13):1218-29. doi: 10.1056/NEJMoa1008234
33. NICE. Obesity: identification, assessment and management. In: Excellence NIfHaC, ed., 2014.
34. NICE. Non-alcoholic fatty liver disease (NAFLD): National Institute for Health and Care Excellence; 2016 [Available from: https://cks.nice.org.uk/topics/non-alcoholic-fatty-liver-disease-nafld/#:~:text=Alcohol%20consumption%20of%20less%20than,cut%2Doff%20to%20diagnose%20NAFLD. accessed 14 December 2020.
35. Augustin S, Pons M, Maurice JB, et al. Expanding the Baveno VI criteria for the screening of varices in patients with compensated advanced chronic liver disease. *Hepatology* 2017;66(6):1980-88. doi: 10.1002/hep.29363 [published Online First: 2017/07/12]
36. Khadjesari Z, White IR, McCambridge J, et al. Validation of the AUDIT-C in adults seeking help with their drinking online. *Addict Sci Clin Pract* 2017;12(1):2. doi: 10.1186/s13722-016-0066-5 [published Online First: 2017/01/05]
37. Saghaei M, Saghaei S. Implementation of an open-source customizable minimization program for allocation of patients to parallel groups in clinical trials. *J Biomed Sci Eng* 2011;4:734-39. doi: 10.4236/jbise.2011.411090
38. Altman DG, Bland JM. Treatment allocation by minimisation. *BMJ* 2005;330(7495):843. doi: 10.1136/bmj.330.7495.843
39. Clark L, Fairhurst C, Torgerson DJ. Allocation concealment in randomised controlled trials: are we getting better? *BMJ* 2016;355:i5663. doi: 10.1136/bmj.i5663 [published Online First: 2016/11/20]
40. EC. Commission Directive 2007/29/EC on foods intended for use in energy-restricted diets for weight reduction. In: Commission E, ed.: European Commission, 2007.
41. Jebb SA, Astbury NM, Tearne S, et al. Doctor Referral of Overweight People to a Low-Energy Treatment (DROPLET) in primary care using total diet replacement products: a protocol for a randomised controlled trial. *BMJ Open* 2017;7(8):e016709. doi: 10.1136/bmjopen-2017-016709 [published Online First: 2017/08/07]
42. Mancia G, Fagard R, Narkiewicz K, et al. 2013 ESH/ESC guidelines for the management of arterial hypertension: the Task Force for the Management of Arterial Hypertension of the European Society of Hypertension (ESH) and of the European Society of Cardiology (ESC). *Eur Heart J* 2013;34(28):2159-219. doi: 10.1093/eurheartj/eht151 [published Online First: 2013/06/19]
43. Brown M, Sinacore DR, Binder EF, et al. Physical and performance measures for the identification of mild to moderate frailty. *J Gerontol A Biol Sci Med Sci* 2000;55(6):M350-5. doi: 10.1093/gerona/55.6.m350 [published Online First: 2000/06/08]
44. Reuben DB, Siu AL. An objective measure of physical function of elderly outpatients. The Physical Performance Test. *Journal of the American Geriatrics Society* 1990;38(10):1105-12. doi: 10.1111/j.1532-5415.1990.tb01373.x [published Online First: 1990/10/01]
45. Guralnik JM, Simonsick EM, Ferrucci L, et al. A short physical performance battery assessing lower extremity function: association with self-reported disability and prediction of mortality and nursing home admission. *J Gerontol* 1994;49(2):M85-94. doi: 10.1093/geronj/49.2.m85 [published Online First: 1994/03/01]
46. Wang CW, Lebsack A, Chau S, et al. The Range and Reproducibility of the Liver Frailty Index. *Liver Transpl* 2019;25(6):841-47. doi: 10.1002/lt.25449 [published Online First: 2019/03/19]
47. Bachtiar V, Kelly MD, Wilman HR, et al. Repeatability and reproducibility of multiparametric magnetic resonance imaging of the liver. *PLoS One* 2019;14(4):e0214921. doi: 10.1371/journal.pone.0214921 [published Online First: 2019/04/11]

48. Reeder SB, Sirlin CB. Quantification of liver fat with magnetic resonance imaging. *Magn Reson Imaging Clin N Am* 2010;18(3):337-57, ix. doi: 10.1016/j.mric.2010.08.013 [published Online First: 2010/11/26]
49. Koutoukidis DA, Beeken RJ, Manchanda R, et al. Diet and exercise in uterine cancer survivors (DEUS pilot) - piloting a healthy eating and physical activity program: study protocol for a randomized controlled trial. *Trials* 2016;17(1):130. doi: 10.1186/s13063-016-1260-1 [published Online First: 2016/03/12]
50. Dennis A, Kelly MD, Fernandes C, et al. Correlations Between MRI Biomarkers PDFF and cT1 With Histopathological Features of Non-Alcoholic Steatohepatitis. *Front Endocrinol (Lausanne)* 2020;11:575843. doi: 10.3389/fendo.2020.575843 [published Online First: 2021/02/16]
51. Li G, Taljaard M, Van den Heuvel ER, et al. An introduction to multiplicity issues in clinical trials: the what, why, when and how. *Int J Epidemiol* 2017;46(2):746-55. doi: 10.1093/ije/dyw320 [published Online First: 2016/12/28]

21. APPENDIX A: STUDY FLOW CHART

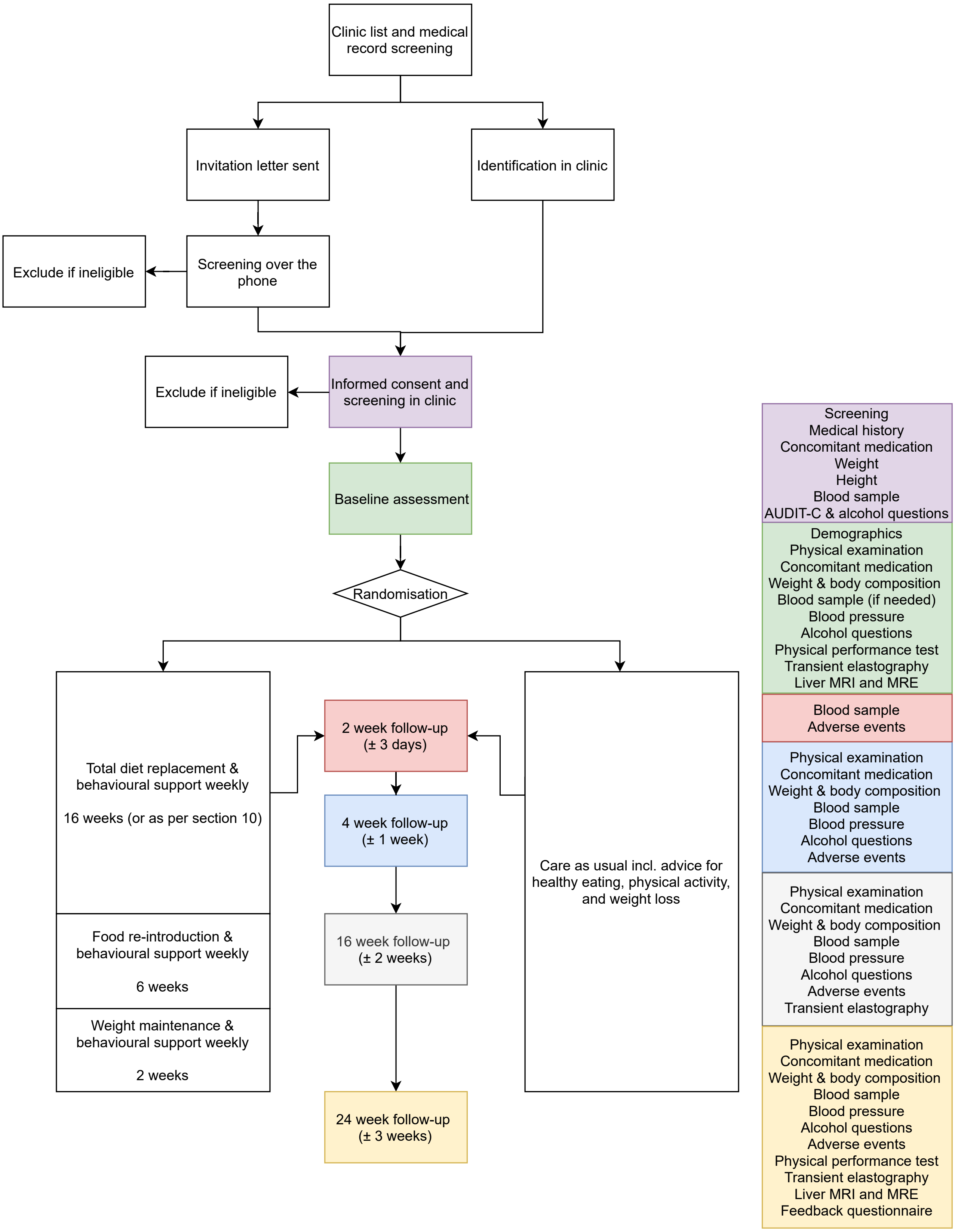

22. APPENDIX B: SCHEDULE OF STUDY PROCEDURES

	**Week -1**	**Week 0**	**Week 2** (± 3 days)	**Week 4** (± 1 week)	**Week 16** (± 2 weeks)	**Week 24** (± 3 weeks)	**Ad-hoc visit**
	Screening	Baseline	Follow-up 1	Follow-up 2	Follow-up 3	Follow-up 4	
Brief interview for eligibility check	x						
Informed consent	x						
Demographics		x					
Medical history	x						
Concomitant medications	x	x		x	x	x	
Physical examination		x		x	x	x	
Weight and body composition	x	x		x	x	x	
Height	x						
Randomisation		x[1]					
ALT, AST, ALP, bilirubin, FBC, INR, PT, U&E	x	x*	x	x	x	x	
C-peptide	x[2]						
HbA1c	x	x*			x	x	
Lipid profile	x	x*				x	
Blood pressure		x		x	x	x	
Physical performance test		x				x	
ELF test	x	x*				x	
Liver MRI / MRE [3]		x				x	
Transient elastography		x			x	x	
AUDIT-C	x						
Alcohol intake questions	x	x		x	x	x	
Adverse event assessments			x	x	x	x	x
Feedback questionnaire						x	

[1] Only after completion of the baseline visit and final eligibility check.

[2] Only for patients with type 2 diabetes who are on insulin for more than 10 years.

[3] MRE only in sites with relevant infrastructure.

* Only if screening blood sample is > 28 days old.

2-year follow-up through medical records: The type and timing of relevant data to be collected will depend on data availability based on each participant's routine clinical care. This is likely to include weight, laboratory results, and health status.

APPENDIX C: STRUCTURE AND CONTENT OF THE INTERVENTION

Figure (Appendix C): Screenshots of the Oviva App

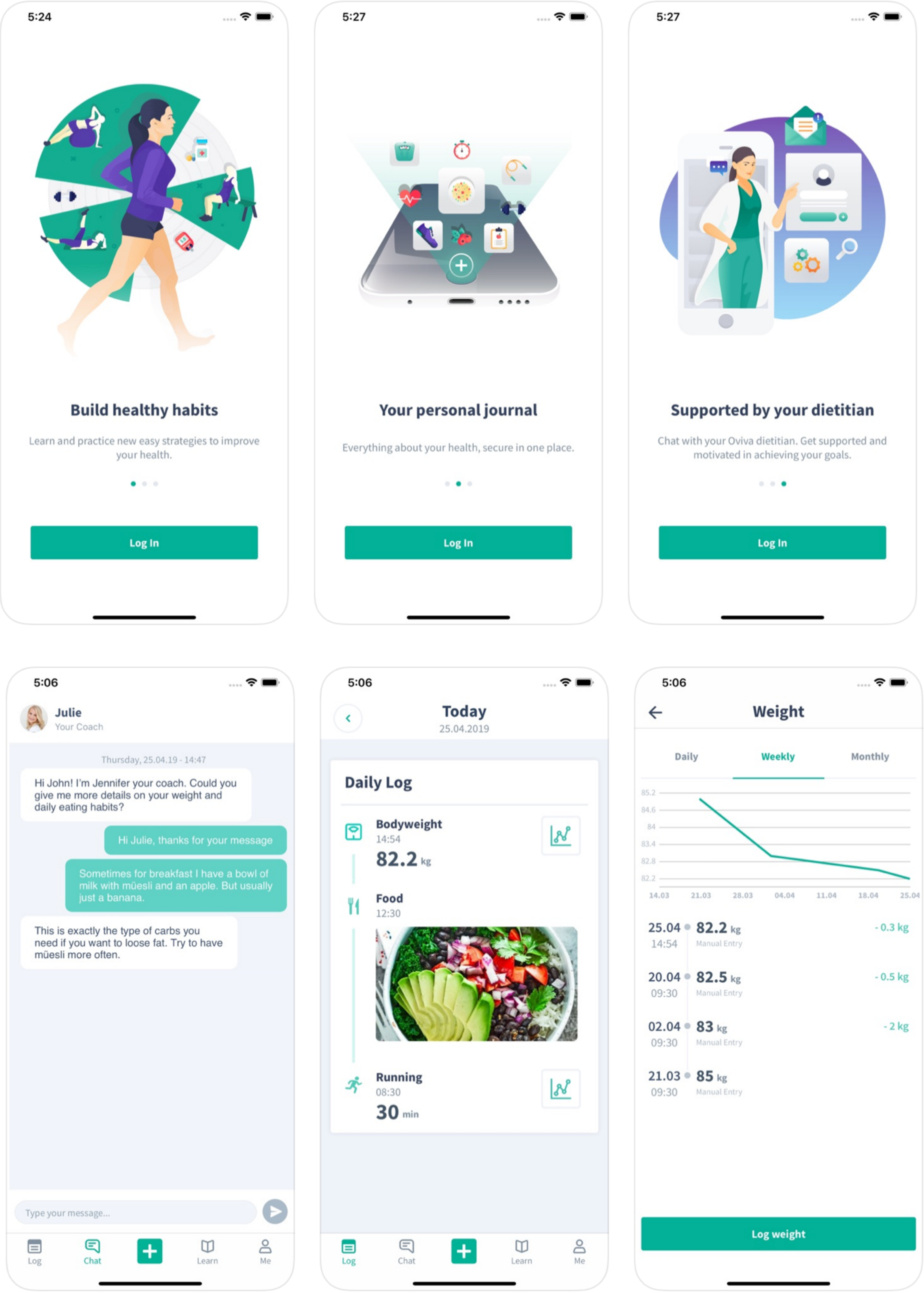

Table (Appendix C): Structure and Content of the intervention

Week	Appointment	Length	Learning topics and key learning outcomes
0	**Initial Consultation**	45 minutes	Initial consultation:<br>Introduces dietitian supporting patient and gives overview of programme,<br>Collects patient history and current medications, assesses motivation and current lifestyle including diet, lifestyle and exercise, problem solves potential barriers and provides education for the TDR stage.<br><br>After their Initial Consultation the patient will be able to access the modules:<br><br>Module 1: Introduction to the Programme<br>Module 2: Stage 1 – the Very Low Calorie Diet<br>Module 3: Stage 1 – resources
1 -16	**Stage 1 – TDR**	15 minutes per week (or 30min fortnightly).<br><br>(either app coaching x3 times per week or phone call)	Reviewing weekly progress include weight change and any issues with blood pressure and blood glucose if needed / monitored.<br>Check product tolerance and usage of fibre supplement.<br>Problem solve any issues encountered.<br><br>Module 4 topics for discussion each week include:<br>Tips for mixing shakes and adding flavour<br>Coping with hunger<br>Managing social situations<br>Problem solving issues and barriers<br><br>At the end of Stage 1, modules 5 and 6 will open to support participant through this next stage of the programme, 4 weeks of food reintroduction<br><br>Cover progress summary to close stage 1:<br>What went well?<br>What were the challenges?<br>Total weight loss<br>Blood glucose review if relevant<br>Blood pressure review if relevant<br>Initial goals review<br>Explanation of Phase 2 and when to stop fibre supplements<br>Signpost to meal plan resources
17-22	**Stage 2 – Food re-introduction**	15 minutes per week (or 30min fortnightly).<br><br>(either app coaching x3 times per week or phone call)	Reviewing weekly progress include weight change and any issues with blood pressure and blood glucose. Look at deviations from dietary protocols and problem solve as needed.<br><br>Coaching utilises these two modules during Stage 2:<br><br>Module 5: Stage 2 – Food reintroduction – including guidance on healthy eating, resetting healthy habits, and reintroducing exercise.

			Module 6: Stage 2 – Food reintroduction resources including meal plans for each increment of calorie increases.<br><br>To close stage 2, progress review undertaken including achievements, challenges, weight lost, changes in blood glucose or blood pressure if relevant, assessing against patient's initial goals and an explanation of Phase 3 with guidance on the calculated energy requirements for phase 3
23-24	**Stage 3 – Sustain and exit call**	15 minutes per week (or 30min fortnightly).<br><br>(either app coaching x3 times per week or phone call)	Coaching calls to work with each participant to create and maintain a diet and lifestyle structure / routine to sustain weight loss and liver health. These review progress over previous month, break down any issues / barriers / lapses, using behavioural change strategies throughout and ensure ongoing self-monitoring and goal setting continues. Coaching utilises following modules at the beginning of this time period:<br><br>Module 7: Sustain including embedding new habits, alcohol, strategies for weight maintenance, avoiding and managing relapse, optimising long term health.<br>Module 8: Sustain resources including meal plans.<br><br>Exit call at week 24: Undertake progress review of the last 6 months including achievements, challenges, changes in dietary choices, volumes and patterns, revisiting initial and evolved goals and expectations, reviewing available outcomes, planning ahead with future goal setting and signpost to local services.

23. APPENDIX D: ENHANCED OBSERVATION ALGORITHM

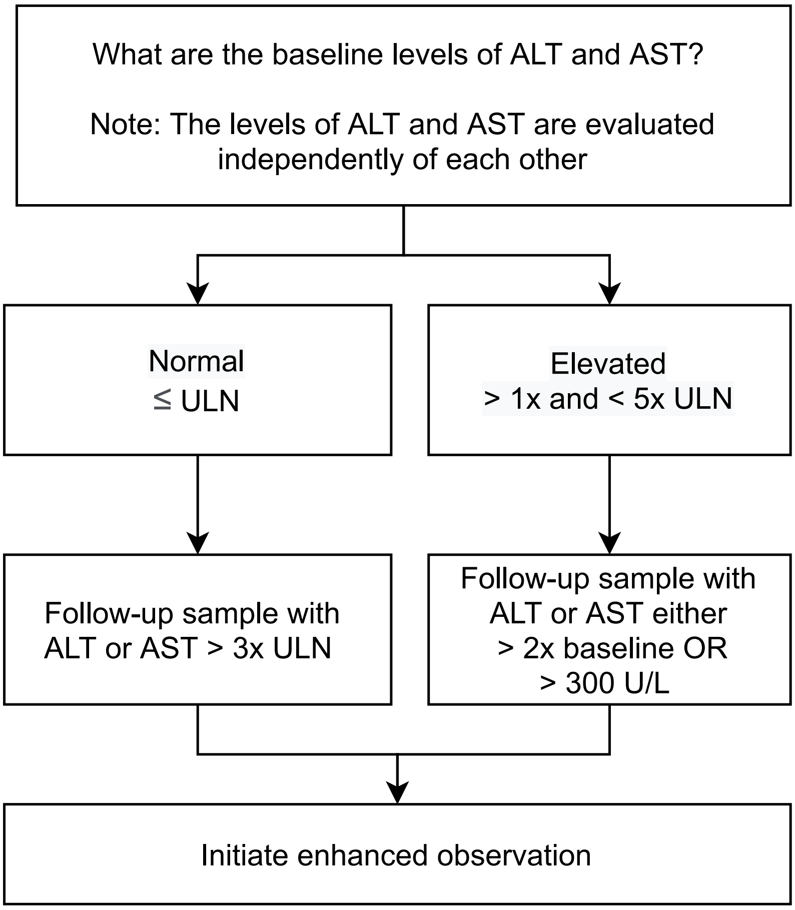

Abbreviations:
ALT: Alanine transaminase
AST: Aspartate transaminase
ULN: Upper limit of normal

24. APPENDIX E: INTERVENTION DISCONTINUATION ALGORITHM

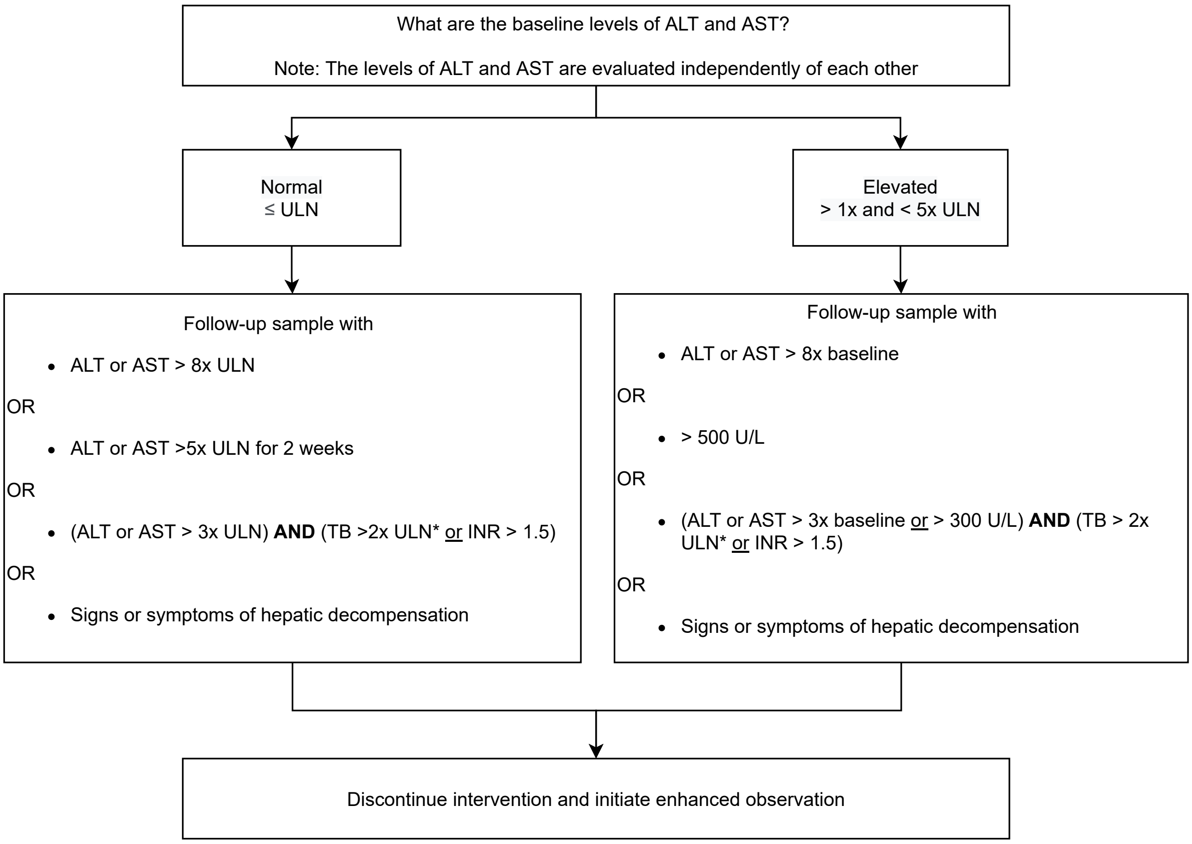

* Unless the participant has Gilbert's syndrome in which case a conjugated bilirubin > 2x baseline will be used instead of total bilirubin.

Abbreviations:
ALT: Alanine transaminase
AST: Aspartate transaminase
ULN: Upper limit of normal
TB: Total bilirubin
Signs or symptoms of hepatic decompensation: jaundice, ascites, hepatic encephalopathy, or variceal haemorrhage.

25. APPENDIX F: AMENDMENT HISTORY

Amendment No.	Protocol Version No.	Date issued	Author(s) of changes	Details of Changes made
1	2.0	20-Dec-21	Dimitrios Koutoukidis	1. Update of REC reference number<br>2. Removal of the NHS number from the data transferred to Oviva<br>3. Amending the intervention frequency from 15 minutes weekly to “15 minutes weekly or 30 minutes fortnightly” to allow flexibility for participants (e.g., in case of holidays).
2	2.1	03-Feb-22	Dimitrios Koutoukidis	1. Addition of ISRCTN number<br>2. Minor wording changes in sections 3 and 6 for consistency<br>3. Clarification of variceal haemorrage as an event of decompensation.
3	2.2	22-Feb-22	Dimitrios Koutoukidis	1. Clarification on the balance test to match the original publication.
4	3.0	23-Feb-22	Dimitrios Koutoukidis	1. Addition of hand-grip strength measurement to allow for calculation of an additional marker of physical performance: the liver frailty index.

5	4.0	11-Jul-22	Dimitrios Koutoukidis	1. Change of exclusion criteria to allow patients with type 2 diabetes on insulin.
6	5.0	17-Aug-22	Dimitrios Koutoukidis	2. Rewording of exclusion criterion for clarity from "no evidence of proliferative retinopathy" to "evidence of proliferative retinopathy"
7	6.0	16-Dec-22	Dimitrios Koutoukidis	1. Addition of a participant identification centre
8	7.0	19-Dec-22	Dimitrios Koutoukidis	1. Addition of 2 more research sites.<br>2. Clarification that MRE will happen only in sites with relevant infrastructure.<br>3. Changes to the exclusion criteria to allow the broadening of inclusion criteria for patients with low-risk varices and those without harmful alcohol consumption.
9	7.1	12-May-23	Dimitrios Koutoukidis	4. Addition of an investigator

List details of all protocol amendments here whenever a new version of the protocol is produced.

Protocol amendments must be submitted to the Sponsor for approval prior to submission to the REC committee and HRA (where required).
